# A Novel Hydroxylation Step in the Taxane Biosynthetic Pathway: A New Approach to Paclitaxel Production by Synthetic Biology

**DOI:** 10.3389/fbioe.2020.00410

**Published:** 2020-05-13

**Authors:** Raul Sanchez-Muñoz, Edgar Perez-Mata, Lorena Almagro, Rosa M. Cusido, Mercedes Bonfill, Javier Palazon, Elisabeth Moyano

**Affiliations:** ^1^Departament de Ciències Experimentals i de la Salut, Universitat Pompeu Fabra, Barcelona, Spain; ^2^Secció de Fisiologia Vegetal, Facultat de Farmacia, Universitat de Barcelona, Barcelona, Spain; ^3^Departamento de Biología Vegetal, Facultad de Biología, Universidad de Murcia, Murcia, Spain

**Keywords:** taxane hydroxylase, paclitaxel, protoplasts transfection, cytochrome P450, biotechnological production, biotransformation

## Abstract

Engineered plant cell lines have the potential to achieve enhanced metabolite production rates, providing a high-yielding source of compounds of interest. Improving the production of taxanes, pharmacologically valuable secondary metabolites of *Taxus* spp., is hindered by an incomplete knowledge of the taxane biosynthetic pathway. Of the five unknown steps, three are thought to involve cytochrome P450-like hydroxylases. In the current work, after an in-depth *in silico* characterization of four candidate enzymes proposed in a previous cDNA-AFLP assay, TB506 was selected as a candidate for the hydroxylation of the taxane side chain. A docking assay indicated TB506 is active after the attachment of the side chain based on its affinity to the ligand 3′*N*-dehydroxydebenzoyltaxol. Finally, the involvement of TB506 in the last hydroxylation step of the paclitaxel biosynthetic pathway was confirmed by functional assays. The identification of this hydroxylase will contribute to the development of alternative sustainable paclitaxel production systems using synthetic biology techniques.

## Introduction

Due to their diversity and wide range of activities, plant secondary metabolites represent a vast source of pharmacological molecules. This group of metabolites are mainly used by plants for defense and environmental interactions and, for that reason, they are produced in very low quantities under normal conditions (Fura, 2006; Breitling et al., 2013). Plant cell cultures are increasingly being applied as biofactories to produce metabolites of pharmaceutical interest (Ramachandra Rao and Ravishankar, 2002; Sharma et al., 2014). These alternative production systems can be manipulated to improve yields by empirical or rational approaches, or ideally a combination of both (Moyano et al., 2003; Vidal-Limon et al., 2018). Yet the metabolic engineering of cell lines is challenged by an incomplete knowledge of highly complex biosynthetic pathways involving numerous enzymes (Zhang et al., 2004).

Taxanes, secondary metabolites produced by *Taxus* spp., are a treatment of choice for several types of cancer (Exposito et al., 2010). This importance lies in the fact that paclitaxel and other taxanes play a dual role in their anticancer effect, a mechanism only shown by this group of compounds. First, they bind and stabilize the dimers of α- and β-tubulin preventing microtubules dynamism and cell division (Magnani et al., 2009). In addition, it has been demonstrated that taxanes bind and inhibit the function of the human protein Bcl-2, involved in preventing apoptosis, enhancing apoptosis and increasing anticancer effects (Fang et al., 1998; Rodi et al., 1999). Due to taxanes limited production by their natural source (concentrations below 0.02% in the inner bark of adult trees), their biotechnological production has become their main current source (Vidal-Limon et al., 2018). *Taxus* spp. cell cultures have yielded higher quantities of paclitaxel and other taxanes than complete plants, and production has been improved by elicitation with methyl jasmonate or coronatine (CORO) increasing their production between 6- and 83-times and their release to the culture medium up to 100% (Cusido et al., 2002, 2014; Expósito et al., 2009; Sabater-Jara et al., 2010; Onrubia et al., 2013; Vidal-Limon et al., 2018). Yields could be improved still further by the engineering of genetically modified lines, but this is hindered by the gaps in our knowledge of the taxane biosynthetic pathway.

Although taxane accumulation varies among species, the biosynthetic pathway is thought to be universal throughout the *Taxus* genus (Cameron and Smith, 2008). To date, 14 of the postulated 19 steps of the pathway have been elucidated (Vongpaseuth and Roberts, 2007). In an initial step, the taxane tricyclic skeleton is constructed from the substrate geranyl-geranyl diphosphate, and after several chemical modifications involving cytochrome-P450 hydroxylases, acyl transferases and other enzymes, the intermediate baccatin III is obtained. The late steps of the pathway include the binding of the side chain by the enzyme baccatin III 13-*O*-(3-amino-3-phenylpropanoyl) transferase (BAPT), the hydroxylation of the side chain at carbon 2′ by an uncharacterized hydroxylase, and benzoylation by 3′-*N*-debenzoyl-2′-deoxytaxol-*N*-benzoyltransferase (DBTNBT), leading to the end product paclitaxel (Croteau et al., 2006).

The limited knowledge of the genes involved in the pathway means taxane biosynthesis remains an open research field. The functional assays required to confirm the suitability of uncharacterized candidate genes can be challenging, but the valuable insights afforded by a computational approach increase their feasibility. This may include the homology evaluation of enzymes that perform similar functions, structural prediction of the putative enzyme, and the performance of docking assays to predict if the candidate enzyme can interact with its putative substrate (Dukka, 2017). Using a combined *in silico* and experimental approach, our group recently identified a gene encoding a novel CoA ligase that activates ß-phenylalanine to form the taxane side chain (Ramírez-Estrada et al., 2016). The gene was characterized using data obtained from a cDNA-amplified fragment-length polymorphism assay (cDNA-AFLP) combined with the metabolic profiling of elicited *Taxus baccata* L. cell cultures (Ramírez-Estrada et al., 2016).

**FIGURE 1:**
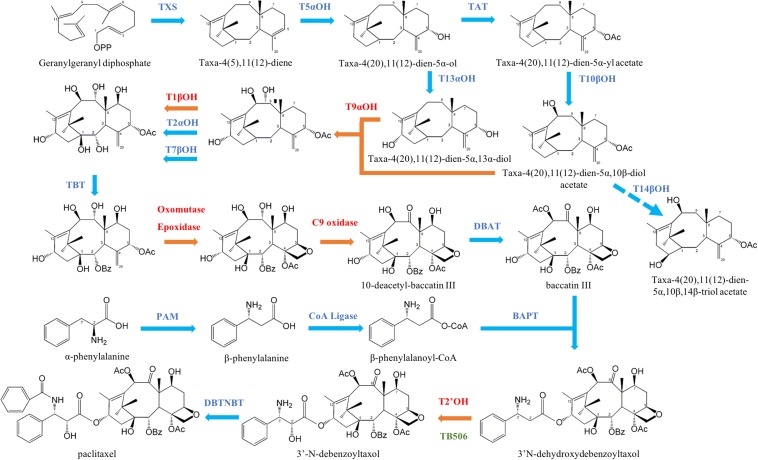
Taxane biosynthesis summary. Enzymes involved in the pathway are in blue if characterized and in red if unknown. Taxadiene synthase (TXS), taxane 5α-hydroxylase (T5αOH), taxadien-5α-ol-*O*-acetyl-transferase (TAT), taxane 13α-hydroxylase (T13αOH), taxane 10β-hydroxylase (T10βOH), taxane 14β-hydroxylase (T14βOH), taxane 2α-hydroxylase (T2αOH) and taxane 7β-hydroxylase (T7βOH), taxane 2α-*O*-benzoyl transferase (TBT), 10-deacetyl-baccatin III-10-*O*-acetyltransferase (DBAT), phenylalanine aminomutase (PAM), 13-*O*-(3-amino-3-phenylpropanoyl) transferase (BAPT), 3′-*N*-debenzoyl-2′-deoxytaxol-*N*-benzoyltransferase (DBTNBT). The candidate hydroxylase TB506 is indicated in the putative site of action.

Among the unknown enzymes involved in the taxane biosynthetic pathway, the hydroxylases are the most numerous group. There are nine hydroxylation steps, but only six hydroxylases have been characterized to date [taxane 5α-hydroxylase (T5αOH), taxane 13α-hydroxylase (T13αOH), taxane 10β-hydroxylase (T10βOH), taxane 14β-hydroxylase (T14βOH), taxane 2α-hydroxylase (T2αOH) and taxane 7β-hydroxylase (T7βOH)] (Jennewein et al., 2001, 2003, 2004; Schoendorf et al., 2001; Chau and Croteau, 2004; Chau et al., 2004; Figure 1).

All the identified hydroxylases of *Taxus* spp. belong to the cytochrome P450 superfamily (subfamily CYP725) and are classified as class II (Kaspera and Croteau, 2006), the most common class in eukaryotes. These enzymes are formed by proteins associated with the outer face of the endoplasmic reticulum, where they are anchored in the membrane with independent NADPH-reductases (Kaspera and Croteau, 2006). Primary structure conservation among plant cytochromes P450 is typically low (ranging from 10 to 15%), but the sequence identity (SID) of taxane cytochrome P450 hydroxylases in *Taxus cuspidata* L. was shown to be higher than 70%, thus indicating a closer relation (Jennewein et al., 2004). This unusual cytochrome P450 group could be the result of a strong sequence conservation associated with a strict substrate selectivity and recognition. Also, although sequence conservation in this enzyme superfamily is low overall, secondary and tertiary structures maintain high conservation rates, which could be the basis for the definition of novel cytochromes P450 (Werck-Reichhart et al., 2002).

Among the known steps of taxane biosynthesis, the most challenging to elucidate have been the side chain attachment to baccatin III and its posterior modifications. The conversion of α-phenylalanine to β-phenylalanine (Walker et al., 2004), the activation of the latter to a CoA ester (Ramírez-Estrada et al., 2016) and its subsequent transfer to baccatin III to form the side chain (Walker et al., 2002) are well established, but it remains unclear whether the 2′-hydroxylation of the β-phenylalanine side chain occurrs before or after its binding. The conversion of the intermediate β-phenylalanoyl baccatin III to 3-phenylisoserinoyl baccatin III in *Taxus* microsomes indicates that the hydroxylation may take place after the amino phenylpropanoid transfer to baccatin III (Long and Croteau, 2005). However, the hydroxylase responsible for this step is still unknown, owing to the complexities of selecting putative hydroxylases belonging to the cytochrome P450 superfamily for functional studies. As well as the difficulty of choosing a suitable candidate, there is a lack of intermediate compounds in the taxane biosynthetic pathway to carry out specific functional assays.

The functional analysis of cytochromes P450 has numerous obstacles: the size of this superfamily, the high degree of divergence among the enzymes, their low transcript abundance and their selective expression. Another limiting factor is their possible need for colocalized electron transfer partners such as NADPH-reductases (Schuler and Werck-Reichhart, 2003). Considering the challenging nature of the functional definition of novel P450 proteins, it is necessary to carry out an exhaustive preliminary study to choose a suitable candidate, thereby overcoming the first of the aforementioned stumbling blocks.

In a previous cDNA-AFLP assay, which provided a wide transcriptome of *Taxus* × *media* cells under MeJA elicited conditions, unknown upregulated proteins were selected as possible candidates on the basis of their putative functions (Onrubia et al., 2014; Ramírez-Estrada et al., 2016). Four of them were suitable to be responsible for some of the hydroxylation steps in paclitaxel biosynthesis. Since further analysis were necessary to reveal the real implication of these candidates in the pathway, in the present study the four putative hydroxylases were studied. The most suitable candidate was selected after an in-depth *in silico* analysis and comparison with the previously characterized taxane hydroxylases. After the construction and optimization of a structural model, the tertiary structure of the selected candidate and the other taxane hydroxylases was studied. Finally, the affinity of the putative hydroxylase with different ligands was studied by a docking assay to confirm its implication in the taxane biosynthetic pathway. Once the suitability of the selected candidate was established, a biotransformation assay was performed within a heterologous system expressing the candidate enzyme and the last enzymes involved in taxane biosynthesis (BAPT and DBTNBT) to verify if the combination of the three enzymes would allow the transformation of intermediate metabolite baccatin III into the final product paclitaxel.

## Results and Discussion

### Candidate Selection and Primary Structure Study

Four of the entire set of candidate enzymes found in previous cDNA-AFLP assays in *T. baccata* (Onrubia et al., 2014; Ramírez-Estrada et al., 2016) were postulated as possible taxane hydroxylases (tagged as TB506, TB224, TB331, and TB574) (Supplementary Table S1). Two of the selected tags (TB331 and TB574) showed homology with known taxane hydroxylases and other putative cytochromes P450, one (TB506) was homologous with a cinnamate 4-hydroxylase (C 4-OH), and the other (TB224) only showed homology with a putative cytochrome P450.

Based on the unusually high sequence identity of the candidates TB331 and TB574 with the characterized taxane hydroxylases, they seemed suitable candidates for the hydroxylases acting on carbons 1β and 9α in the intermediate steps of the taxane pathway, hydroxylating the taxane core. In contrast, TB506 showed a low amino acid sequence conservation with the characterized taxane hydroxylases, as could be expected for the hydroxylase acting on the *N*-benzoyl-3-phenylisoserinoyl side chain at C2′. Furthermore, the structural similarity of the substrates of the unknown C2′ hydroxylase and C 4-OH could explain the homology shown by these two enzymes (Supplementary Figure S1). Despite this preliminary analysis seems indicate that TB506 could be a hydroxylase acting on the C2′ of taxane structure, further sequence and structural features were studied to clarify its implication in taxanes biosynthesis.

Since all the characterized hydroxylases belong to the superfamily cytochrome P450, the unknown hydroxylases were also expected to belong to this protein group. The identification of novel cytochrome P450 proteins involves studying the sequences and structural traits related to differential features (Goyal et al., 2015). First, sequence identities (SIDs) between the characterized hydroxylases and the putative taxane hydroxylase TB506 were analyzed to identify conserved and variable sequence regions.

**FIGURE 2:**
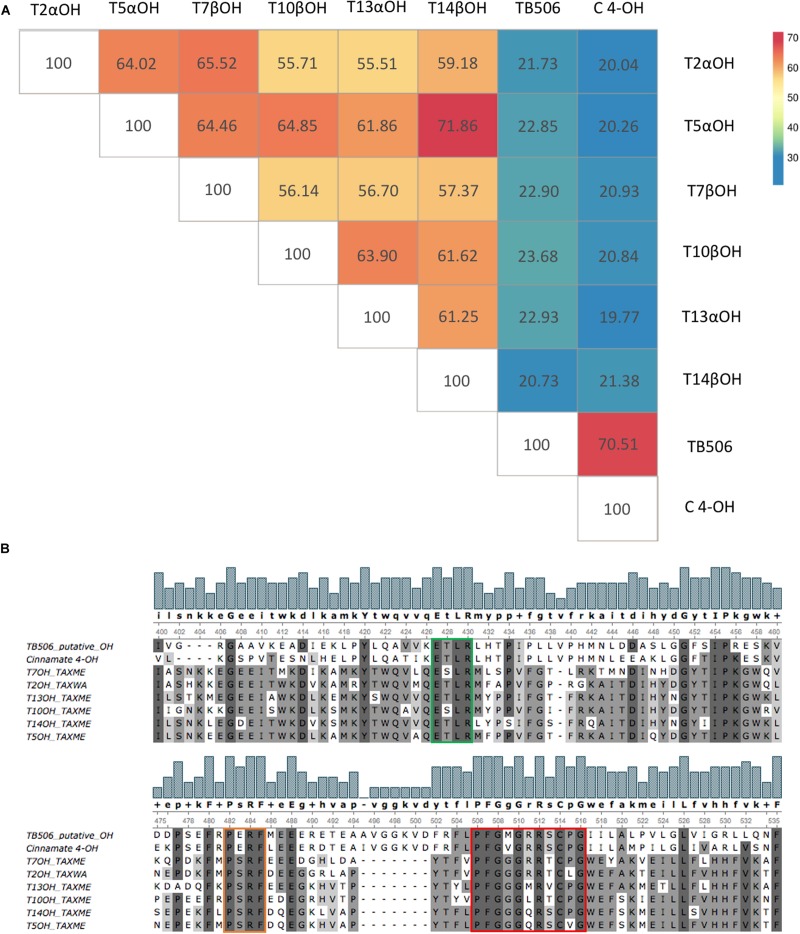
Primary sequence alignment between taxane hydroxylases T2αOH, T5αOH, T7βOH, T10βOH, T13αOH, T14βOH, the candidate TB506 and the cinnamate 4-hydroxylase (C 4-OH). **(A)** Matrix similarity constructed by SIDs. Color scale ranges from blue (the minimum value of sequence identity) to red (the maximum value of sequence identity). **(B)** Sequence alignment. Consensus sequences are highlighted in green (the salt bridge ETLR involved in the stabilization of the enzyme core), orange (the PERF motif which interacts with the ETLR motif and locks the heme-binding group) and red (the heme-binding group that serves as a ligand for the cofactor heme-iron).

Thus, a primary structure comparison was performed among the characterized taxane hydroxylases in an intraspecific alignment (belonging to a single *Taxus* species). Although no *Taxus* species has a complete set of known hydroxylases, the high level of homology between species allowed us to add sequences from another organism (Jennewein et al., 2004). Despite relatively high SIDs, ranging from 55.51 to 71.86% (Figure 2), two clusters of genes were found: T10βOH, T13αOH, and T14βOH in one, and T2αOH and T7βOH in the other. In contrast, T5αOH showed high SID with all the hydroxylases, especially with T14βOH. These results reflect the different action sites of the hydroxylases. T10βOH, T13αOH, and T14βOH hydroxylate the taxane core in the initial steps of the pathway, which starts with the hydroxylation of taxadiene-5α-ol and taxadiene-5α-yl acetate by T10βOH and T13αOH, respectively. The biosynthesis of taxanes then continues or the activity of T14βOH leads to the formation of taxadiene-5α,10β-14β-triol acetate, a derivative compound not included in the pathway (Kaspera and Croteau, 2006). Situated in the intermediate steps are T2αOH and T7βOH, which were observed clustered together. Involved in the early steps of the pathway, T5αOH is able to hydroxylate both tautomers produced by the action of taxadiene synthase (TXS), indicating lower substrate selectivity. This higher substrate permissiveness could explain the higher SIDs shown by T5αOH in comparison with the other hydroxylases.

When the candidate TB506 was added to the comparison, its alignment showed a SID of 20.73 and 23.68% with T14βOH and T10βOH, respectively, clearly lower than the SID between the characterized hydroxylases. The sequence similarity of the known hydroxylases, depending on their hydroxylation site, matched with the low SID of the candidate TB506 with them, which could be explained by its putative function hydroxylating the side chain. When added to the comparison, C 4-OH was found to share a 75.51% SID with TB506, whereas lower SIDs were obtained with the other hydroxylases, with values similar to those of TB506 (Figure 2A).

A primary structure comparison also allowed us to detect conserved features between the different enzymes. Some signature sequences shared among cytochromes P450 are involved in common mechanisms, such as electron and proton transfer or the activation of the oxygen molecule to carry out the hydroxylation reaction (Bak et al., 2011): the heme-binding group with the consensus sequence ([FW]-[SGNH]-X-[GD]-{F}-[RKHPT]-{P}-C-[LIVMFAP]-[GAD]) containing the essential cysteine residue, the ETLR motif with the absolute conserved E-X-X-R pattern and the consensus sequence PERF motif (Du et al., 2016).

Figure 2B shows the sequence alignment of the characterized taxane hydroxylases, the candidate TB506 and the characterized C 4-OH. Despite the low SID between the hydroxylases and TB506 (Figure 2A), CYP450 signature consensus patterns were found in all analyzed sequences, confirming membership of the same superfamily. The typical PERF sequences were only observed in TB506 and C 4-OH, while the variation PSRF was found in the other hydroxylases, in accordance with their unusually high degree of conservation (Jennewein et al., 2004). The ETLR motif exhibited the absolute conserved E-X-X-R pattern in all cases, while the other two variable residues were present in all the studied sequences with the exception of T7βOH and T10βOH. The heme-binding group matched the consensus patterns of the CYP450 superfamily in all the studied sequences, and the variable amino acids of the candidate TB506 matched some of the characterized taxane hydroxylases.

Accordingly, despite the low SID between the candidate TB506 and the other taxane hydroxylases, the presence of the consensus sequences of the salt-bridge ETLR, the heme-binding group and the PERF motif indicate that all the tested enzymes belong to the same superfamily. This constitutes further evidence that the candidate TB506 functions as a hydroxylase in the taxane biosynthetic pathway (Bak et al., 2011; Du et al., 2016). Furthermore, since the motifs PERF, ETLR and the heme-binding group are involved in protein folding, the higher aminoacid conservation in these motifs between TB506 and C 4-OH are the first indicative of their binding site similarity.

### Structural Model Building and Geometric Optimization

**FIGURE 3:**
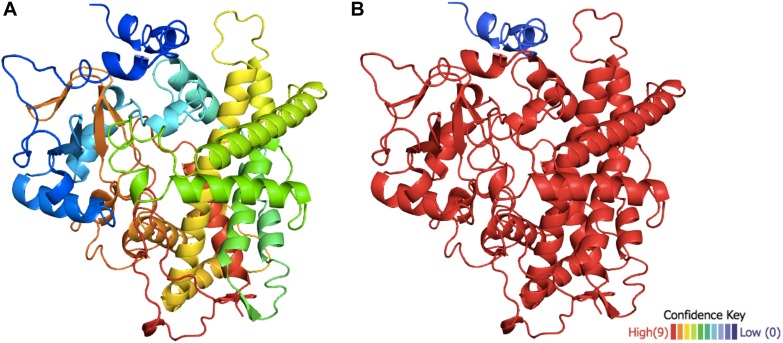
Structural model of the putative hydroxylase TB506. **(A)** Predicted tertiary structure of TB506. **(B)** The confidence level is shown on a scale from >90 to <10% (red to blue, respectively).

Due to the low primary sequence conservation observed in the previous alignments, secondary and tertiary structures were also studied. As no structural information about any taxane hydroxylase was available, predictions were performed using all the enzymes in the previous analysis, as described above. To increase the reliability of the predicted structures, different oxidoreductases belonging to the same CYP superfamily from different organisms were used as templates the complete table of templates for TB506 is available in the Supplementary Table S1 and the templates for the rest of structures in Supplementary Table S2. In the case of the putative hydroxylase TB506, a model was achieved with a confidence level of >90% in 95% of the molecule (Figure 3), showing a low confidence level only at the N-terminal α-helix.

After modeling the putative TB506 hydroxylase, the knowledge-based energy pattern of the molecule was studied to detect the erroneous parts of the model (Wiederstein and Sippl, 2007; Cossio et al., 2012). The knowledge-based pattern showed two high peaks of positive knowledge-based energy at the N-terminal sequence, and a central lower peak and a moderate one at the C-terminal sequence (Supplementary Figure S2A), indicating a structural problem. Thus, a geometric optimization was necessary to find the conformation with minimal potential energy and avoid errors in side chain packaging or van der Waals clashes between atoms. After an energy minimization and structural optimization to relax the structure and avoid van der Waals clashes between atoms, some of the peaks reduced their knowledge-based energy, resulting in unmodified high peaks in the N-terminal (residues 1–40 of the model), in the central part (residues 275–301) and in the C-terminal (residues 430–451) (Supplementary Figure S2B). The rest of the hydroxylase models showed a knowledge-based energy pattern very similar to the putative TB506 hydroxylase. As shown in Supplementary Figure S2C, the first problematic region contains an alpha-helix, while the other two are external loops.

To reveal the cause of the high knowledge-based energy found in the N-terminal sequence, the subcellular location of the protein was predicted to check if some of these points are accomplishing specific functions. As expected, the prediction showed that the high peak found in the N-terminus corresponds, with a high confidence degree, to a transmembrane helix that anchors the protein to the membrane and situated the rest of the molecule in the cytoplasm (Supplementary Figure S3). Furthermore, the subcellular location prediction situated the putative hydroxylase (TB506) in the secretory pathway with high confidence (0.884), possibly being anchored in the endoplasmic reticulum membrane by the transmembrane helix detected previously. This situates the putative hydroxylase TB506 in class II of the cytochrome P450 superfamily, like the characterized taxane hydroxylases (Kaspera and Croteau, 2006). The rest of the regions with high peak energies did not show any probability to be membrane-associated zones, so when defining the regions interacting with the substrate it will be necessary to study if they are involved in the substrate recognition.

### Structural Traits of the Putative Hydroxylase TB506

**FIGURE 4:**
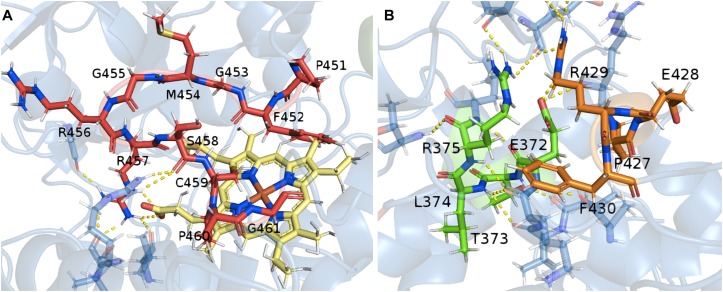
Relationship between the conserved motifs in the putative hydroxylase TB506. **(A)** Consensus heme-binding group motif (red) and the heme-group (yellow). **(B)** Interactions between the PERF (orange) and ETLR (green) motifs with the protein core. In yellow, the hydrogen bond interaction.

Thanks to the model of the confirmed hydroxylase TB506, the role of the previously identified consensus sequences could be studied in the structure. The CYP450 signature motif responsible for the heme-ligand bond was observed interacting with the heme-group by several hydrogen bonds (Figure 4A). The motifs PERF and ETLR, which stabilize the structure, were found to be connected by hydrogen bonds and also linked with other surrounding residues to form a stable protein core, thus establishing the E-R-R triad (Figure 4B). These features and their correct orientation and interactions confirmed that the model of the candidate has a correct folding, allowing it to be used in the docking assay.

To detect structural conservation, the structures of TB506, the characterized taxane hydroxylases and the C 4-OH were compared by a structural alignment (Supplementary Table S3). The alignment of the candidate TB506 with the C 4-OH showed the lowest root-mean standard deviation (RMSD) value, being the enzyme with the highest structural similarity with our putative hydroxylase. The set of characterized taxane hydroxylases showed relatively low RMSD values when aligned with TB506, indicating structural similarity [although previous sequence alignments showed high sequence variations (Figure 2A)].

In order to know if the RMSD values were low enough to indicate similarity between this group and the candidate TB506, an external structure not belonging to the superfamily cytochrome P450, but with the same SID as the characterized hydroxylases, was added to the comparison (1DSB protein, an oxidoreductase from *Escherichia coli*). As expected, the RMSD of the external structure and the candidate TB506 was very high in comparison with the other values, indicating a strong structural divergence. With this result, we can confirm that despite the low primary structure conservation between the characterized taxane hydroxylases and the candidate TB506, they belong to the same cytochrome P450 superfamily, showing their typical high degree of sequence divergence for similar P450 structures (Schuler and Werck-Reichhart, 2003). Furthermore, the higher structural similarity between TB506 and C 4-OH could indicate that they are sharing also structural features that are not present in the characterized taxane hydroxylases, such as the binding site.

### Molecular Docking

The structural conservation between cytochromes P450, especially of the regions involved in substrate recognition, allow the study of relevant structural traits of novel CYPs (Baudry et al., 2003). Thus, we aligned the putative hydroxylase TB506 with the CYP450 prototype (see section “Experimental procedures”) to detect the residues belonging to the solvent channel and the active site, which would be used as coordinates to perform the docking assay to detect ligand interactions. In this alignment, the defined substrate recognition sites (SRS) of the CYP450 prototype were extrapolated to the structure of our sequence, and the involved residues were identified by their structural location (Supplementary Figure S4). All the regions identified as SRSs defined a solvent channel that allows external compounds to arrive to the active site of the CYP450, where the heme-group is situated and interacts with the signature patterns. Since the transmembrane helix does not contribute to the conformation of the protein or to the hydroxylase activity, the apo form of the protein was used to carry out the docking assay. It should be noted that none of the loops showing high knowledge-based energy (Supplementary Figure S2) were involved in the SRSs, so they would not interfere in the docking assay.

The previous assays had confirmed that the candidate TB506 belongs to the cytochrome P450 superfamily, but there were still no data about its possible interaction with potential ligands. To detect if there are interactions between the ligands of T2′OH and the candidate, a docking assay was performed. Since cytochrome P450 proteins are characterized by an extensive plasticity and flexibility in their active site (Hritz et al., 2008), an ensemble of 220 alternative conformations was created by molecular dynamics (MD) simulations before launching the docking process, as described in Pang and Kozikowski (1994).

By the construction of the 220 structural variants, different orientations of the side chains were obtained and, thereafter, temperature and structural fluctuations that could affect the solvent channel volume and size are taken into account, representing the typical flexibility of CYP450 binding sites. ß-phenylalanine, ß-phenylalanine-CoA and 3′*N*-dehydroxydebenzoyltaxol structures were then docked into the complete set of structures as possible ligands (Supplementary Figure S5).

From the entire set of results, the ligand energies range from -4.0 to -7.0 kcal/mol using ß-phenylalanine as a ligand, from -6.53 to -10.80 kcal/mol with ß-phenylalanine-CoA and from -4.4 to -7.5 kcal/mol with 3′*N*-dehydroxydebenzoyltaxol. The more negative the ligand energies, the more affinity was shown for the receptor; and all the results showed good interactions between the candidate and all the ligands assayed. Despite this, not all the docking results were correctly situated in the active site. Thus, the complete set of results was screened to consider only the docking outputs situating the ligand near the active site.

**FIGURE 5:**
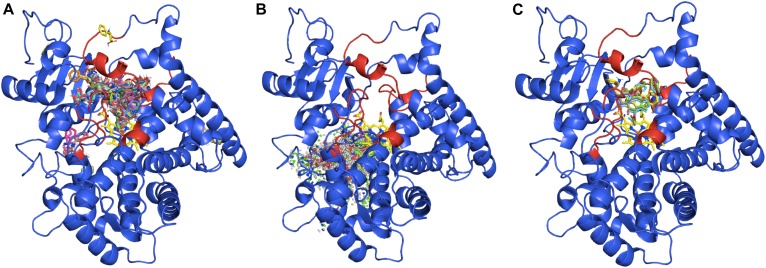
Docking outputs for the candidate TB506 and the ligands ß-phenylalanine **(A)**, ß-phenylalanine-CoA **(B)** and 3′N-dehydroxydebenzoyltaxol **(C)**. The regions responsible for the recognition of the substrate are highlighted in red.

Taking into account all the outputs from the docking assay, ß-phenylalanine is almost always inside the solving channel and near the heme-group (Figure 5A), whereas ß-phenylalanine-CoA is never inside the active site (Figure 5B), due to its bigger size. The 3′N-dehydroxydebenzoyltaxol docking assay only gave five results in the expected position (Figure 5C), possibly because the larger structure of this ligand can only fit in the active site in a few structural variants of the candidate TB506. In addition, these outputs situate the ligand interacting with the residues involved in the previously defined SRSs, indicating the correct binding of 3′*N*-dehydroxydebenzoyltaxol to the active site (Supplementary Figure S6). The best of these five results was -7.4 kcal/mol, which is a better result than the complete set obtained with ß-phenylalanine. For this reason, although both ligands seem to be interacting with the putative hydroxylase TB506, the complete structure of 3′*N*-dehydroxydebenzoyltaxol seems to be a better ligand than ß-phenylalanine, supporting a hydroxylation role in the side chain of the taxane biosynthetic pathway. The possible interaction with two of the ligands assayed indicates that, like other taxane hydroxylases such as T5αOH (Croteau et al., 2006), our candidate may have affinity for different structures involved in taxane biosynthesis. In the same way, the action of the putative hydroxylase TB506 on the substrate ß-phenylalanine-CoA can be discarded due to structural incompatibilities, with no docking result situating the ligand in the active site.

Therefore, the results of the *in silico* characterization indicate that the candidate TB506 is a hydroxylase belonging to the CYP450 superfamily and the docking assay revealed high affinity with the ligand 3′*N*-dehydroxydebenzoyltaxol, situating it in the hydroxylation step after BAPT activity, as expected.

### Biotransformation of Baccatin III to Paclitaxel

**FIGURE 6:**
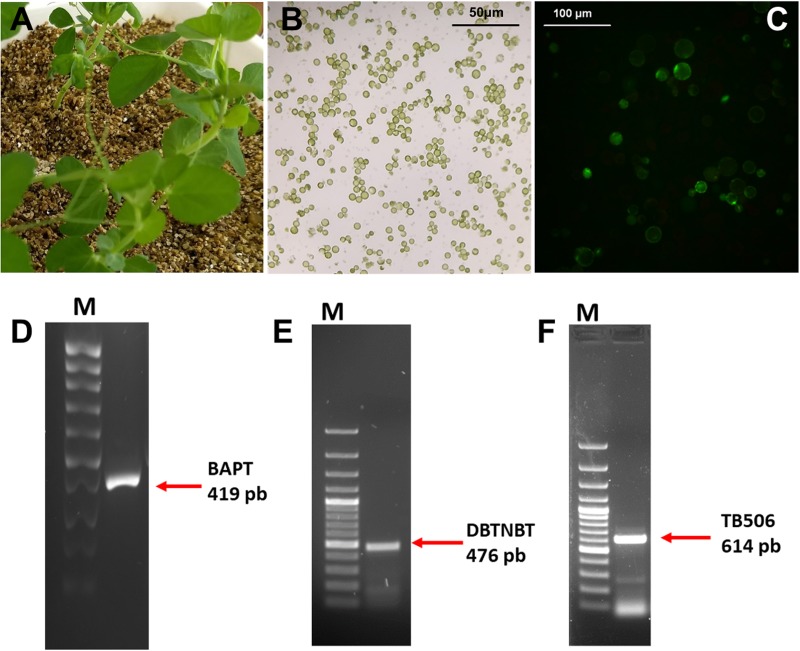
Source plant material **(A)**, protoplast culture **(B)** and cell viability **(C)** (assessed by DAF as described in section “Materials and Methods”) of transfected *P. sativum* protoplasts treated for 6 h with 1 μM coronatine. RT-PCR analysis of BAPT **(D)**, DBTNBT **(E)** and TB506 **(F)** genes in *P. sativum* protoplasts at 6 h of treatment with coronatine. First lanes of the gels correspond to a 100-bp plus DNA ladder.

Once the suitability of the selected candidate was demonstrated by the exhaustive *in silico* characterization, a functional assay was performed with the hydroxylase TB506 using a heterologous system of *Pisum sativum* L. protoplasts (Figures 6A–C). Thus, protoplasts of *P. sativum* were transiently transfected with the genes BAPT, DBTNBT and TB506, and then incubated with 1 μM CORO for 6 h in the presence of 100 mg/L baccatin III and 100 mg/L β-phenylalanine-CoA. As shown in Figure 6C, *P. sativum* protoplasts remained viable after the treatment with CORO and both paclitaxel precursors (with about 90% viability). In agreement with these results, Ketchum et al. (2007) reported that the addition of 125 μM (70.82 mg/L) baccatin III to *Taxus* cells increased paclitaxel production and the cells remained viable.

In order to detect the transgenes expression, a RT-PCR were performed to confirm the transfection process as described in Sanchez-Muñoz et al. (2019). This analysis revealed that BAPT, DBTNBT and the candidate TB506 were expressed in the protoplasts of *P. sativum* under elicitation conditions (Figures 6D–F).

The use of transfected *P. sativum* protoplasts as heterologous system was chosen because the previous results suggested the putative hydroxylase TB506 was a class II CYP450, which colocalize with NADPH-reductases in the endoplasmic reticulum membrane to perform electron transfer and hydroxylation (Schuler and Werck-Reichhart, 2003). As this kind of proteins need to be anchored in the endoplasmic reticulum, *in vitro* functional assays are complex. Therefore, due to the feasibility of transient protoplast transfection (Sheen, 2001), a *P. sativum* protoplast culture was chosen as a suitable heterologous system.

Once the expression of the three genes was verified, protoplasts transiently transfected with the genes BAPT, DBTNBT and the candidate TB506 were incubated in the osmotic medium W5 containing the intermediate metabolites of the taxane biosynthetic pathway ß-phenylalanine-CoA and baccatin III in the presence of 1 μM CORO. In order to differentiate the action of the hydroxylase TB506 from other hydroxylases present in the *P. sativum* protoplasts, which could be acting non-specifically, protoplasts transfected only with BAPT and DBTNBT were added to the analysis as control samples.

Expecting low quantities of paclitaxel in the transfected samples due to the different conditions of the heterologous system, a method using a HPLC-MS/MS analysis that allows the detection of taxane traces was used (Gallego et al., 2015). The HPLC-MS/MS analysis performed after the incubation of the transfected protoplasts for 72 h showed that baccatin III and paclitaxel were present in all analyzed samples, both at the extra- and intracellular level (Supplementary Figure S7). The ion fragmentation pattern of baccatin III was found in all samples (m/z 587.248, m/z 207.101, m/z 405.190, m/z 509.216, m/z 527.227, and m/z 327.164 eluting at 10.10 min) (Supplementary Figure S8), matching with the ion fragmentation of the standard solution of baccatin III (Supplementary Figure S9). These results indicate that part of this intermediate metabolite, both intra- and extracellular, remained unmetabolized by the transfected protoplasts. The presence of intra- and extracellular baccatin III indicates its delivery to the apoplast of the *P. sativum* protoplast after its addition to the osmotic medium. Ketchum et al. (2007) showed that labeled baccatin III molecules were able to enter *Taxus* cells and be transformed into paclitaxel, indicating an ability to cross the plasma membrane and be metabolized by the last enzymes of the taxane biosynthetic pathway. Although baccatin III was found in the protoplasts transfected with the three genes (BAPT, TB506, and DBTNBT) and in the control samples (only transfected with BAPT and DBTNBT), the quantity of baccatin III detected in the samples containing the candidate TB506 was slightly lower (0.0881 g/L in the samples containing the three genes in contraposition of the 0.0999 g/L in the control samples), indicating that part of this compound could had been metabolized.

**FIGURE 7:**
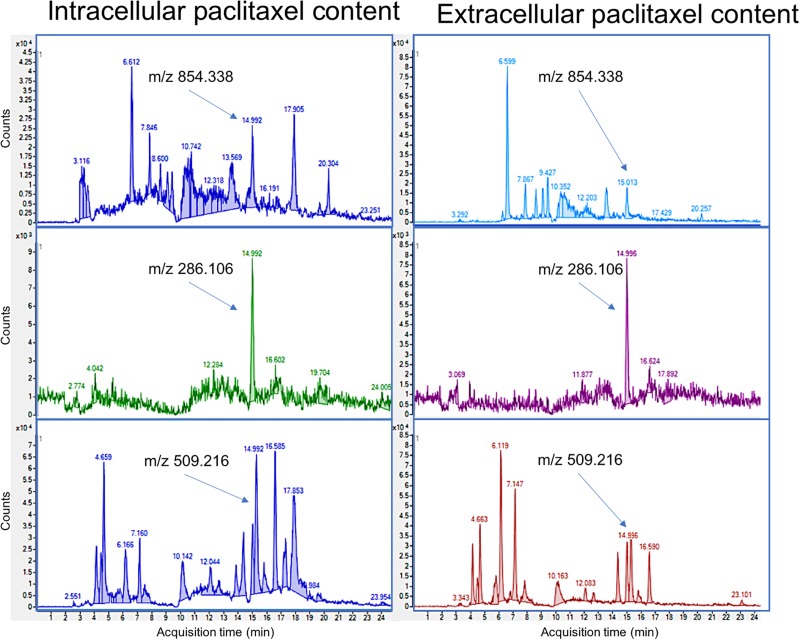
HPLC-MS/MS chromatograms of intra- and extracellular paclitaxel in *P. sativum* protoplasts transiently transfected with the genes BAPT, DBTNBT and the candidate TB506 and treated with 1 μM coronatine in the presence of baccatin III and ß-phenylalanine-CoA. From top to bottom, the chromatograms represent the different molecular ions corresponding to paclitaxel (m/z: 854.338, 286.106, and 509.216 at the retention time of 14.992 min).

In order to know if the lack of baccatin III in the protoplasts expressing the candidate TB506 is due to its conversion to paclitaxel, the presence of this metabolite in the transfected protoplasts was studied. The presence of paclitaxel in the transfected protoplasts was confirmed by its retention time and comparing the MS/MS fragmentation pattern with the standard (ions at m/z 854.338, m/z 509.216 and m/z 286.106 eluting at 14.992 min) (Figure 7 and Supplementary Figure S10). On contrary, the control samples not containing the candidate TB506 lacked this peak, indicating that the presence of the genes BAPT and DBTNBT was not able to transform baccatin III into paclitaxel, pointing out TB506 as the responsible for the needed hydroxylation step. Regarding the exact quantity of paclitaxel found in the studied samples, it was detected 0.00188 and 0.00119 g/L of paclitaxel in the intracellular and extracellular content, respectively. The intra- and extracellular paclitaxel content of the transfected samples indicates that the three transgenes (BAPT, TB506, and DBTNBT) are involved in binding the side chain to baccatin III, side chain hydroxylation, and the last benzoylation step to obtain the end product paclitaxel.

The detection of both intra- and extracellular paclitaxel (as well as baccatin III) suggests a flux allowing its movement across the cell membrane. Paclitaxel secretion in *Taxus* spp. cells is dependent on specific mechanisms requiring ATP consumption (Naill et al., 2012). Sabater-Jara et al. (2014) reported an enhanced expression of a gene encoding an unspecific ABC transporter in *Taxus* cell cultures elicited with cyclodextrins and methyl jasmonate, as well as an increase in extracellular paclitaxel. This indicated that ABC transporters could be involved in the transfer of intracellular paclitaxel to the culture medium (Sabater-Jara et al., 2014). Similarly, our results suggest that ABC-like transporters in *P. sativum* protoplasts can transfer paclitaxel molecules.

To discard the effect of a non-specific hydroxylase encoded by the *P. sativum* genome, control samples expressing only the genes BAPT and DBTNBT were studied. Baccatin III was detected in the control samples as it was added to the protoplast medium as a precursor (Supplementary Figure S11A), but no signals for paclitaxel at the specific retention time or any of the corresponding ions were observed in any of the analyzed samples (Supplementary Figure S11B).

These results confirm that the hydroxylase tagged as TB506, in combination with the enzymes BAPT and DBTNBT, is able to metabolize baccatin III in the presence of the activated precursor of the ß-phenylalanine-CoA side chain, producing paclitaxel as the end product. Accordingly, the gene TB506 encodes a hydroxylase involved in the final hydroxylation step of taxane biosynthesis, and its identification completes the last unknown step at the end of the pathway. The definition of this novel protein, and the method successfully used here to biotransform the valuable intermediate metabolite baccatin III into paclitaxel, opens up possibilities of developing novel strategies for the *in vivo* production of paclitaxel, avoiding the classical chemical semi-synthesis.

## Conclusion

With the aim of enhancing the biotechnological production of paclitaxel, the suitability of four candidate enzymes derived from a previous cDNA-AFLP assay was studied to unravel the unknown hydroxylation steps of taxane biosynthesis. Based on the homology of the putative hydroxylase TB506 with the enzyme cinnamate 4-hydroxylase and the similarity of their substrates, TB506 was selected as a candidate enzyme responsible for the hydroxylation of the ß-phenylalanoyl side chain near the end of the taxane biosynthetic pathway. The primary structure comparison, together with the structural traits in the constructed models, revealed signature cytochrome P450 patterns and a strong structural conservation between the candidate and previously characterized taxane hydroxylases. Thus, the analysis situated TB506 within the cytochrome P450 superfamily, supporting its putative function as a hydroxylase. Moreover, *in silico* analysis proved to be a useful approach to overcome the challenge of selecting suitable candidates for the functional characterization of cytochromes P450.

A favorable interaction between the hydroxylase TB506 and the ligands potentially involved in the last hydroxylation step of the pathway was observed in the docking assay. A subsequent biotransformation assay using baccatin III and the side chain precursor ß-phenylalanine-CoA showed the capacity of the hydroxylase TB506, together with BAPT and DBTNBT, to produce the end product paclitaxel.

The suitability of TB506 for the final hydroxylation step, postulated to occur after the side chain-binding by the BAPT enzyme, is supported by its high affinity with the 3′*N*-dehydroxydebenzoyltaxol structure and the functional assay results. Yet the affinity shown toward the ß-phenylalanine structure indicates a degree of plasticity in substrate recognition, as in previously characterized taxane hydroxylases. The ability of BAPT to transfer the hydroxylated side chain structure was shown experimentally, indicating that the hydroxylase could have residual activity in the side chain before its binding to baccatin III. The affinity for ß-phenylalanine situates this residual activity before the action of the CoA-ligase, as the complete structure with the CoA molecule attached is unable to enter the active site of the hydroxylase TB506.

The definition and characterization of this hydroxylase contribute to the clarification of one of the most elusive steps of the taxane biosynthetic pathway and bring the complete elucidation of paclitaxel biosynthesis closer. This hydroxylation step could be a key factor in the development of environmentally friendly biotechnological approaches to paclitaxel production based on the abundant intermediate baccatin III, commonly used for the chemical semi-synthesis of paclitaxel.

## Experimental Procedures

### Primary Structure Study

The software Clustal Omega (Sievers et al., 2011; Li et al., 2015) was used to generate an amino acid multiple sequence alignment (MSA) between the characterized hydroxylases of several *Taxus* spp.: T5αOH [*T. cuspidata* (Q6WG30), *T.* × *media* (Q6SPR0) and *T. wallichiana* var. *chinensis* (Q8H6A8)]; T13αOH [*T. cuspidata* (Q8W4T9), *T*. × *media* (Q5BU48) and *T. wallichiana* var. *chinensis* (Q56GD5)]; T10αOH [*T. cuspidata* (Q9AXM6), *T.* × *media* (H9BII4) and *T. wallichiana* var. *chinensis* (Q6JLD0)]; T2αOH [*T. wallichiana var. chinensis* (Q5S1U2) and *T. canadiensis* (Q6JD68)]; T14αOH [*T.* × *media* (H9BII0) and *T. cuspidata* (Q84KI1)] and T7αOH [*T*. × *media* (H9BII3), *T. cuspidata* (Q6JTJ0) and *T. mairei* (G8XSN5)]. The different amino acid sequences were aligned to reveal the sequence identity (SID). Furthermore, sequences of a cinnamate 4-OH (C 4-OH) (B2Z6P5) and the candidate TB506 (AKH04263) were added to the MSA. To visualize and analyze the MSA, the software Unipro UGENE v1.29.0 (Okonechnikov et al., 2012) was used. The online tools TMHMM (Krogh et al., 2001) and TargetP 1.1 (Emanuelsson et al., 2000) were used to reveal the subcellular location of the candidate hydroxylase TB506.

### Structural Model Building, Validation and Geometric Optimization

The Phyre2 online tool (Kelley et al., 2015) was used to predict the structural conformation of the characterized taxane hydroxylases, the candidate TB506 and the cinnamate 4-OH (C 4-OH). Different oxidoreductases belonging to the same CYP superfamily from different organisms served as templates (Supplementary Table S1). NAMD software (Phillips et al., 2005) was used to find the optimal conformation of all the models, minimizing the energy of each residue. The process was carried out in two phases, first modifying the orientation of the side chains and then the complete residues, to avoid structural changes due to incorrect sidechain packaging. Two thousand cycles and a dielectric constant of 80 were used. PROSAweb software (Wiederstein and Sippl, 2007) was used to reveal the quality of the prediction and possible problematic regions of the model with high potential energy, both in the original model and the optimized structure.

### Molecular Dynamics and Protein-Ligand Molecular Docking

To define the coordinates of the active site to launch the docking assay, a structural alignment between the candidate TB506 and the CYP450 prototype (PDB code: 2HPD) (Schuler and Werck-Reichhart, 2003) was performed by the PyMOL Molecular graphic system (DeLano scientific: San Carlos, CA, United States). Molecular dynamics (MD) calculations were performed by NAMD software using the apo form of the enzyme containing the heme-group (Phillips et al., 2005). In previous MD calculations, an energy minimization was carried out using standard conditions (10,000 cycles, 1 femtosecond per cycle and a dielectric constant of 80). The MD calculations gave 220 derivative structures from the candidate TB506. Putative substrates were constructed and topologically optimized by ACD/ChemSketch software v1.7 2007 (ACD/Labs: Toronto, ON, Canada). Molecular docking was performed by AutoDockTools 4 (Morris et al., 2009) providing the coordinates corresponding to the active site of the molecule.

### Protoplast Isolation

For protoplast isolation, young leaves of *P. sativum* were cut in 1 mm strips. The enzymatic cocktail consisting of 2% cellulase, 0.1% pectinase from *Rhizopus* spp. and 0.3% macerozyme R10 from *Rhizopus* spp. was added to an osmotic medium (consisting of 10 mM Tris-MES, 0.6 M sorbitol, 1 mM CaCl_2_ and 0.1% BSA at pH 5.4). Then, 2 g of plant material were incubated with 10 ml of digestion medium (the enzymatic cocktail diluted in the osmotic medium at pH 5.4). After incubating the plant material for 1 h, the protoplasts were harvested by filtration with 0.65 μm nylon filters and centrifugation at 100 g for 5 min, before being rinsed and cleaned three times with the same osmotic medium at pH 7. Finally, the protoplasts were resuspended in 2 ml osmotic medium at pH 7 before proceeding to the transfection assay. Protoplasts were quantified under a light microscope with a Neubauer Hemocytometer, using the formula: protoplast yield (protoplasts/ml) = average number of protoplasts counted per section × 10^4^ × dilution factor. Protoplast viability was determined using fluorescein diacetate (DAF) and propidium iodide (IP) as described by Duncan and Widholm (1990).

### Protoplast Transfection Assay

Genes BAPT (KC988329.1), the candidate TB506 (KP178208), and DBTNBT (AY563629.1) were cloned into the plasmids pK7WG2D, pJCV52 (Laboratory of Plant Systems Biology, Ghent University, Belgium), and pCAMBIA1301 (GeneScript, Piscataway, NJ, United States), respectively, and introduced in *E. coli* DH5α. The plasmid was extracted using the PROMEGA Wizard Plus SV Minipreps DNA Purification System Kit. To transfect the protoplasts obtained from *P. sativum*, 10 μg of each plasmid DNA were placed in a 2 ml round-bottom tube and approximately 5 × 10^6^ protoplasts were added and carefully mixed. 1× volume of a PEG solution consisting of 40% PEG6000, 100 mM glucose, 10 mM CaCl_2_, and 0.7 mM KH_2_PO_4_ at pH 5.8 adjusted with KOH was added to the tube dropwise and slightly mixed. After 15 min of incubation, 400 μl of W5 medium (2 mM MES, 154 mM NaCl, 125 mM CaCl_2_ and 5 mM KCl) was added following the same procedure as with the PEG solution, and protoplasts were harvested by centrifugation at 600 *g* for 2 min. Following the same procedure, samples of the obtained protoplasts were transfected only with the genes BAPT and DBTNBT and used as a negative control to discard the effect of unknown hydroxylases on the substrates.

### Biotransformation of Baccatin III to Paclitaxel

Transfected protoplasts were placed in 0.5 ml of W5 medium containing 1 μM CORO, 0.1 g/L baccatin III and 0.1 g/L β-phenylalanine-CoA and they were incubated at 25°C in darkness for 72 h. After the incubation time, cells were harvested by centrifugation at 13,000 rpm for 3 min. Total taxanes were extracted from both cells and culture media (Cusidó et al., 1999; Bonfill et al., 2003) and paclitaxel detection was performed using the method for the detection of taxanes traces by liquid chromatography-tandem mass spectrometry (HPLC-MS/MS) (Gallego et al., 2015).

### Transfection Confirmation

After the incubation with 1 μM CORO, 0.1 g/L of baccatin III and 0.1 g/L of β-phenylalanine-CoA, the expression of the three genes was studied in the transfected protoplasts at 6 h of treatment. Total RNA was extracted from approximately 500 mg of protoplast pellet using the ARNzol kit (REAL, Valencia, Spain) and treated with Superscript IV Reverse Transcriptase (Invitrogen, Carlsbad, CA, United States) and the TURBO DNA-free kit (Thermo Fisher Scientific, Waltham, MA, United States) to obtain complete cDNA from 1 μg of total RNA. cDNA from the transfected protoplasts was amplified with specific primers for BAPT (forward: TGAGCGAGTCATGGTAGAC; reverse: AACCCCCACATGTAAAACGA), TB506 (forward: GGGAACGGGCAAGACATGG; reverse: GCCCACTCCATCGA CCACAG) and DBTNBT (forward: CGGGGGGTTTGTTGTGG GATT; reverse: CATTATCCATTGCACATG) using the Dream-Taq Green PCR Master Mix kit (ThermoFisher Scientific). The PCR conditions were: denaturation at 95°C for 5 min, 35 cycles of 95°C for 1 min, Tm (64°C for BAPT, 58°C for TB506 and 48°C for DBTNBT primers) for 1 min and 72°C for 1min, with a final step at 72°C for 5 min.

## Data Availability Statement

The datasets generated for this study can be found in the Genbank KP178208 (TB506).

## Author Contributions

JP and EM conceived the project and designed the research plan. RS-M performed the computational approaches for the *in silico* characterization. EP-M and LA performed the functional analysis. EM supervised the computational work. RC supervised the functional analysis. RS-M and EM wrote the manuscript. MB, RC, and JP supervised and complemented the writing. RS-M and EP-M contributed equally to this manuscript.

## Conflict of Interest

The authors declare that the research was conducted in the absence of any commercial or financial relationships that could be construed as a potential conflict of interest.
