# Supplementary material for: A Novel Hydroxylation Step in the Taxane Biosynthetic Pathway: A New Approach to Paclitaxel Production by Synthetic Biology
Source: Front Bioeng Biotechnol. 2020 May 13;8:410. doi: 10.3389/fbioe.2020.00410 (PMC7247824; doi:10.3389/fbioe.2020.00410)
Supplement: Supplementary file 1 [file Data_Sheet_1.docx]

Supplementary Material

**Table S1. Hydroxylase candidates in the taxane biosynthetic pathway.** The putative function was extrapolated from the best BLAST hit described in the table.

| **Tag** | **Putative function** | **GenBank accession** | **BLAST hit code** | **Blast hit annotation** | **e-value** |
| --- | --- | --- | --- | --- | --- |
| TB506 | C2’ hydroxylation | KP178208 | B2Z6P5 | Cinnamate 4-hydroxylase | 0.0 |
| TB331 | C1 or C9 hydroxylation | KP178206 | D7T4D5 | Putative cytochrome P450 | 1.0E-118 |
| TB574 | C1 or C9 hydroxylation | KP178209 | D7T4D5 | Putative cytochrome P450 | 1.0E-118 |
| TB224 | Unknown | KP178204 | D5A7Y6 | Putative cytochrome P450 | 1.0E-152 |

**Table S2. Different structures used as templates for the modelling of each protein.**

| **T2αOH** | **T5αOH** | **T7βOH** | **T10βOH** | **T13αOH** | **T14βOH** | **TB506** | **C 4-OH** |
| --- | --- | --- | --- | --- | --- | --- | --- |
| 4LXJ 3K9V 1TQN 3E4E 5T6Q | 4LXJ  3E4E 3NA0 2X2N 5T6Q | 4LXJ 1TQN  3K9V 5T6Q 3E4E | 4LXJ  3E4E 3K9V 1TQN 3NA0 | 4LXJ  3E4E 3K9V 5T6Q 3NA0 | 4LXJ  3E4E 3NA0 2X2N 3K9V | 2HI4 1NR6 2NNJ 3E4E 1PO5 | 3E4E 4LXJ 1PO5 2NNJ 3EBS |

**Table S3. Sequence identity (SID) and root mean standard deviation (RMSD) of the structural alignments between the putative hydroxylase TB506 and the other structures (the taxane hydroxylases T2αOH, T5αOH, T7βOH, T10βOH, T13αOH, T14βOH, the cinnamate 4-hydroxylase (C 4-OH) and an oxidoreductase from *E. coli* (1DSB)).**

|  | **T2αOH** | **T5αOH** | **T7βOH** | **T10βOH** | **T13αOH** | **T14βOH** | **C 4-OH** | **1DSB** |
| --- | --- | --- | --- | --- | --- | --- | --- | --- |
| **SID** | 21.73 | 22.85 | 22.90 | 23.68 | 22.93 | 20.73 | 70.51 | 21.91 |
| **RMSD** | 2.426 | 2.200 | 2.718 | 3.286 | 2.544 | 2.687 | 1.941 | 16.40 |


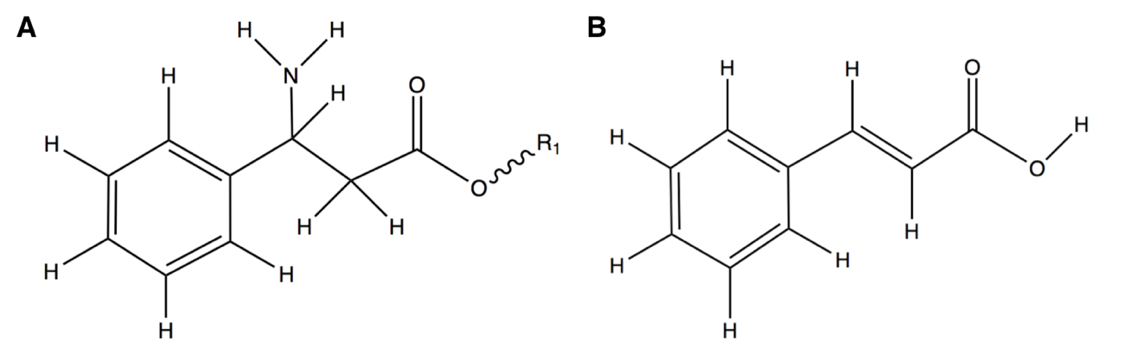


**Figure S1. Structural similarity between the substrates for (A) the putative T2’OH hydroxylase, N-benzoyl-3-phenylisoserinoyl lateral chain, and for (B) the cinnamate 4-hydroxylase, cinnamic acid.**


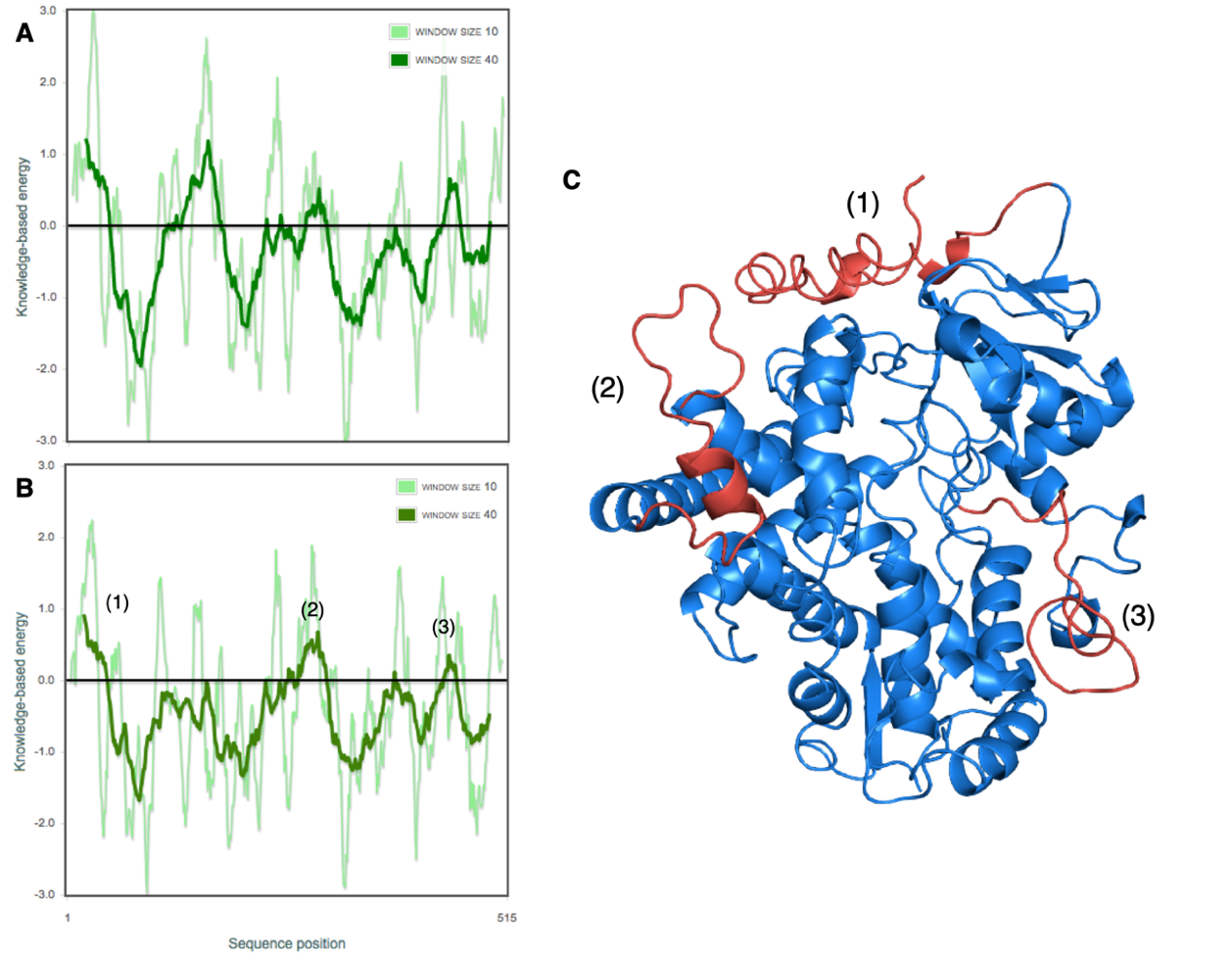


**Figure S2. Knowledge-based energy pattern of TB506 hydroxylase before (A) and after (B) energy optimization.** (C) The zones with high knowledge-based energy are highlighted in red, corresponding to amino acids 1-40 (1), 275-301 (2) and 430-451 (3).

**
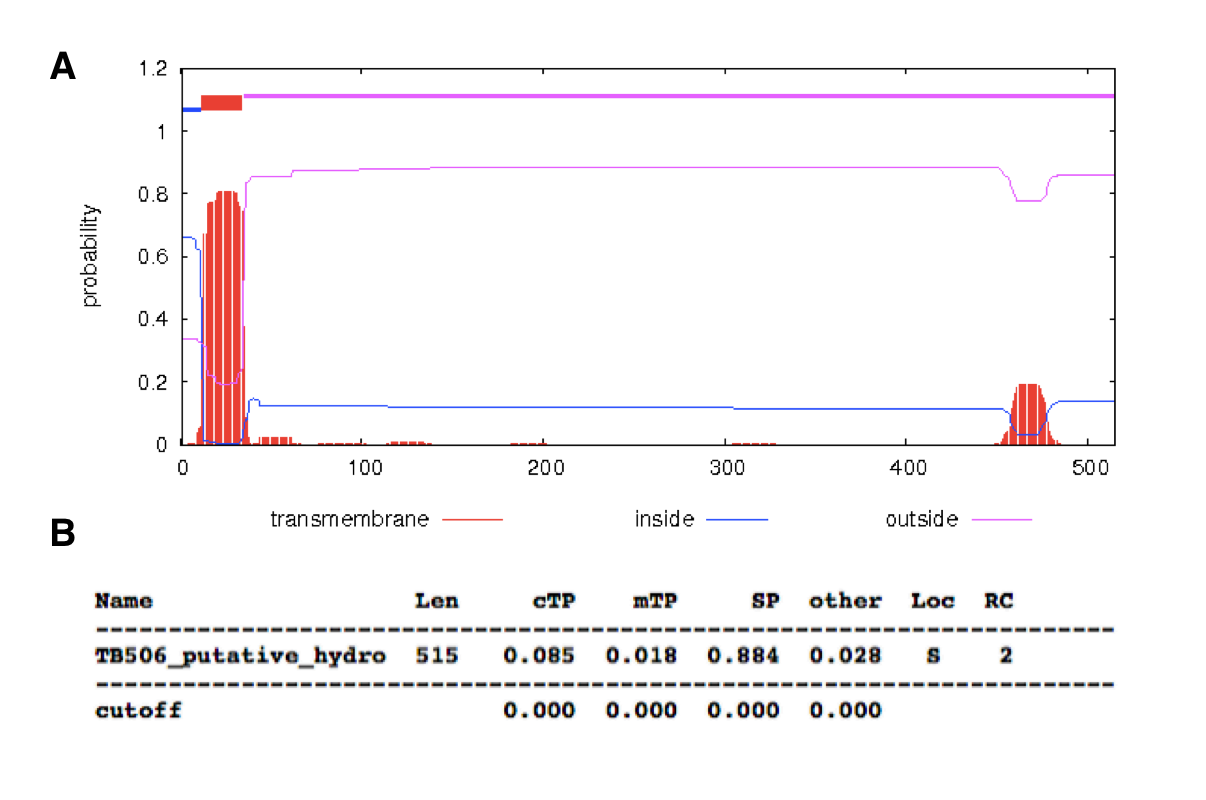
**

**Figure S3. Prediction of the subcellular location of the TB506 hydroxylase.** (A) Transmembrane helix prediction. (B) Subcellular location of the TB506 hydroxylase (cTP: chloroplast transit peptide; mTP: mitochondrial targeting peptide; SP: Signal peptide; S: Secretory pathway; RC: Reliability class; 2: (0.800 > x > 0.600)).


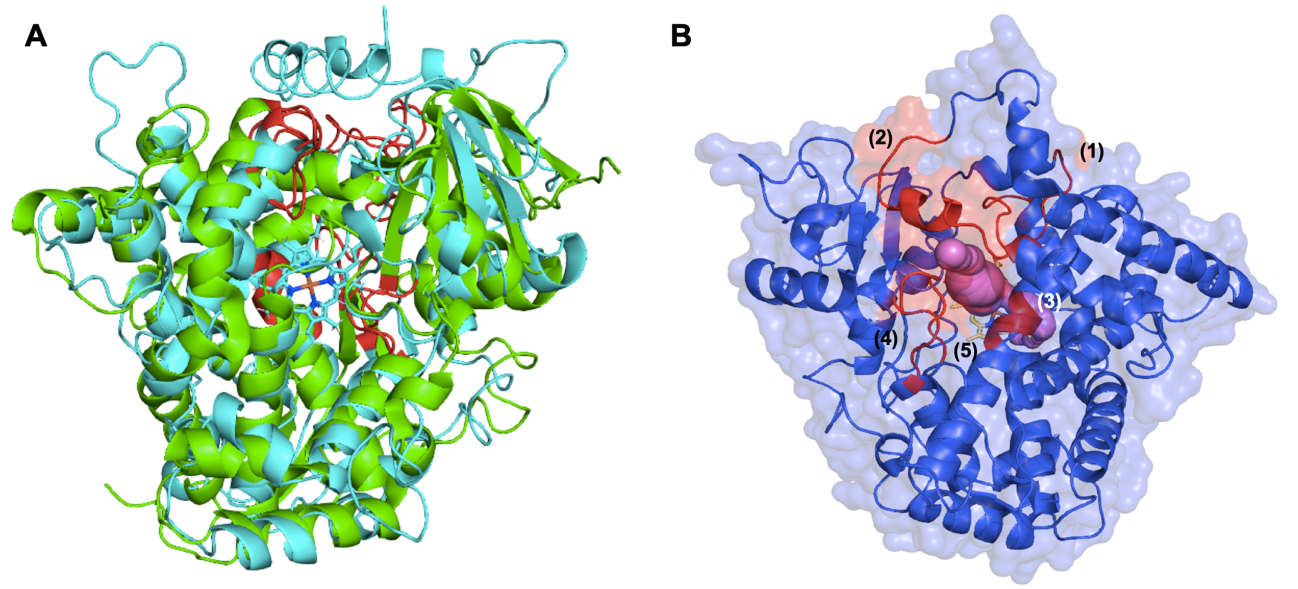


**Figure S4. Substrate recognition sites (SRSs) identified by structural conservation.** (A) Structural alignment between the CYP450 prototype (green) and the putative hydroxylase TB506 (blue). (B) Relation between the substrate recognition sites (SRSs) in the putative hydroxylase TB506 and the solvent channel. In red, the different SRSs are highlighted (1: SRS1 (122-135); 2: SRS2 (226-240); 3: SRS4 (315-319); 4: SRS5 (377-381) and 5: SRS6 (497-503)). The solvent channel is shown in purple.


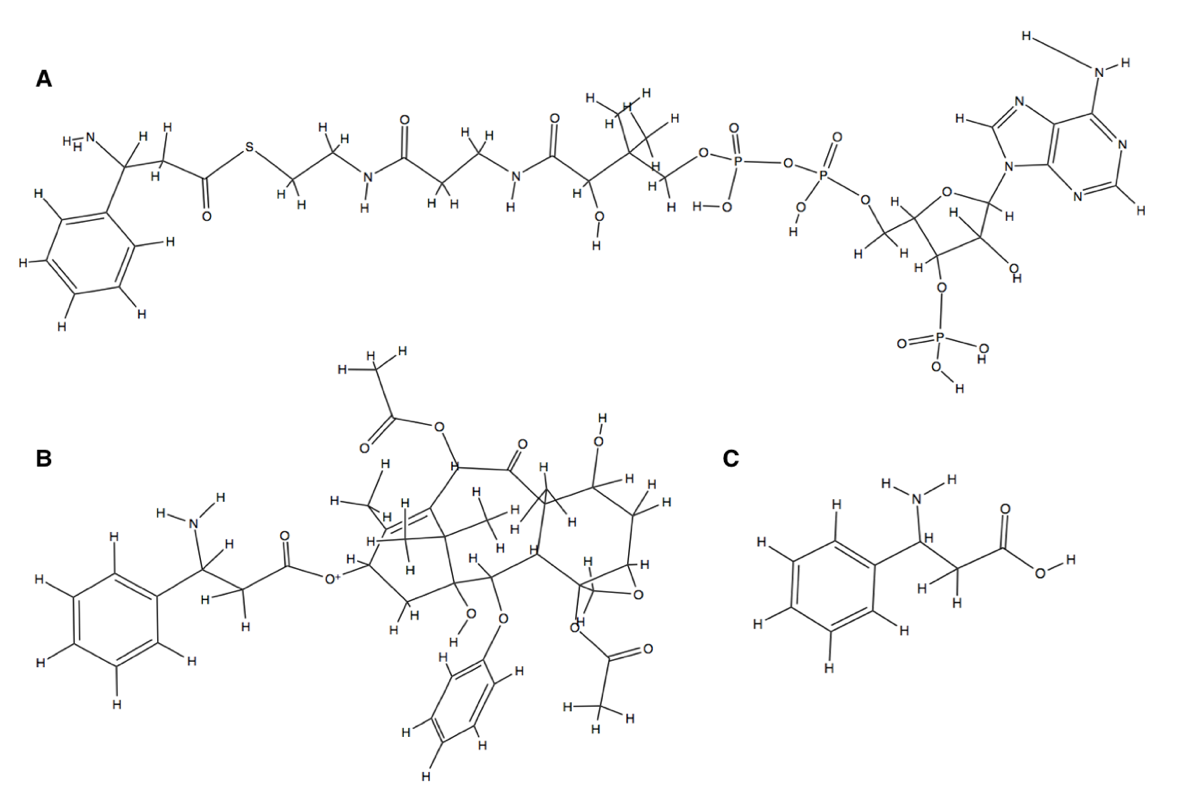


**Figure S5. Set of ligands used in the docking assay**. A) ß-phenylalanine-CoA; B) 3’N-dehydroxydebenzoyltaxol; C) ß-phenylalanine.


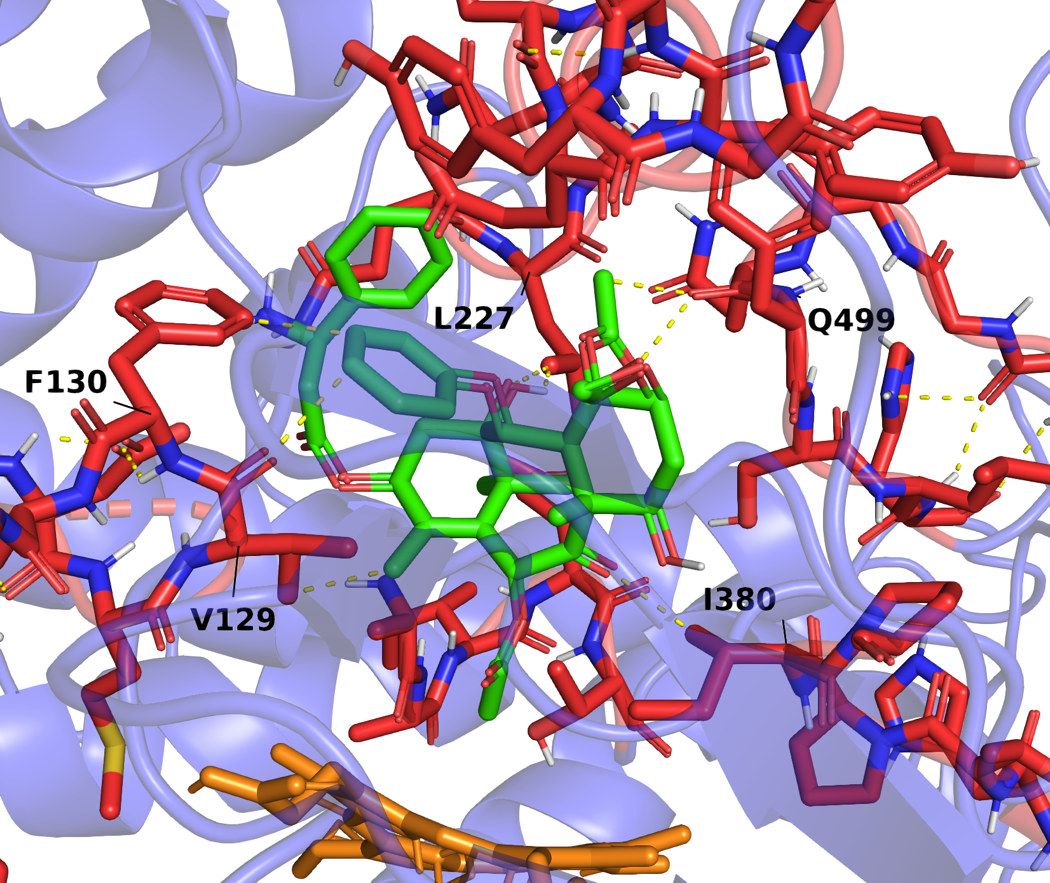


**Figure S6. Docking output for the candidate TB506 (blue) and the ligand 3’N-dehydroxydebenzoyltaxol (orange).** In red, SRSs are highlighted. The heme group is colored in orange.


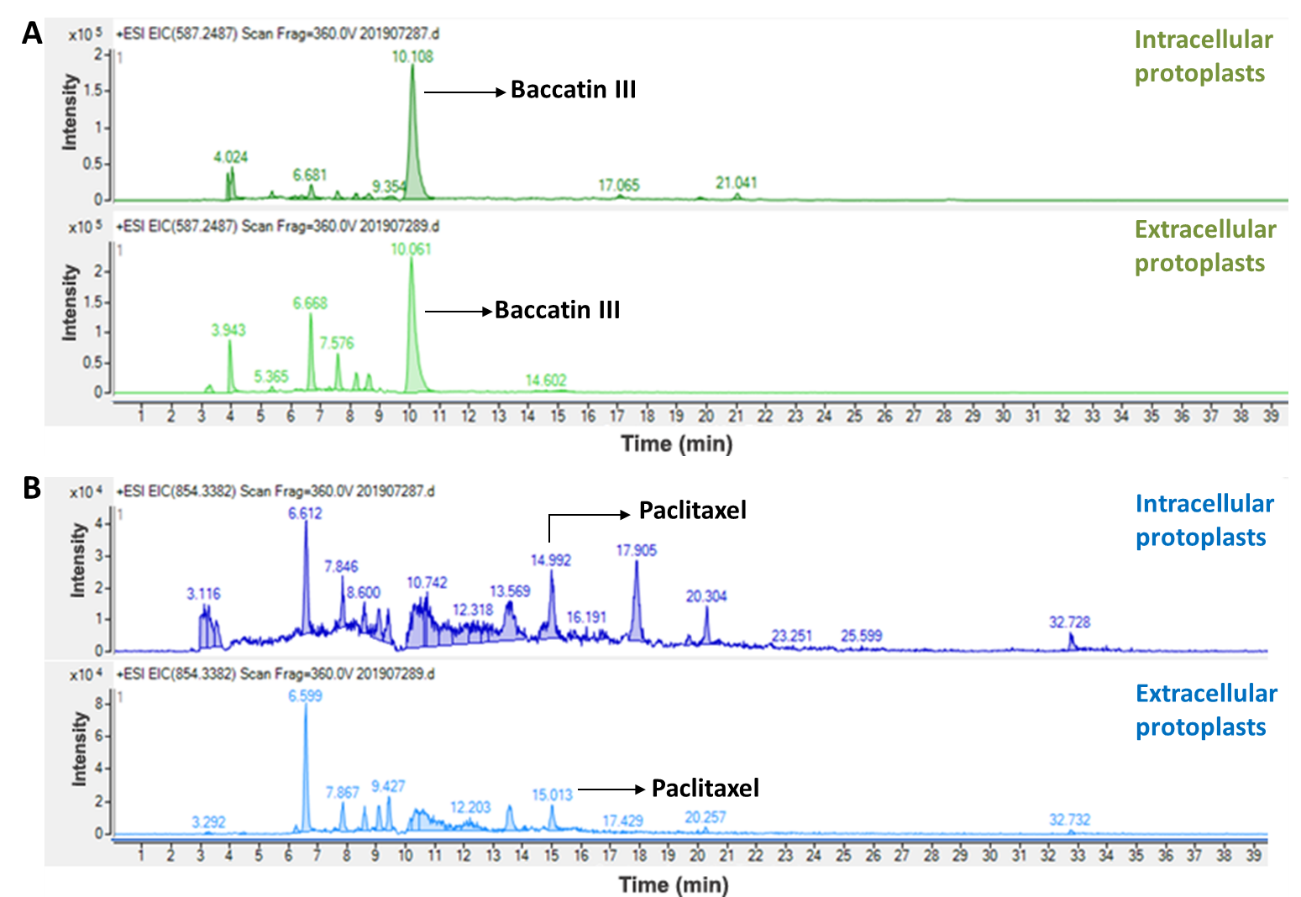


**Figure S7. HPLC-MS/MS analysis of protoplasts transiently transfected with the genes BAPT, DBTNBT and the candidate TB506 treated with 1 µM coronatine in the presence of baccatin III and ß-phenylalanine-CoA.** (A) Identification of baccatin III in both inside and outside protoplasts of P. sativum at 72 h of incubation. (B) Identification of paclitaxel in both inside and outside protoplasts of P. sativum at 72 h of incubation.


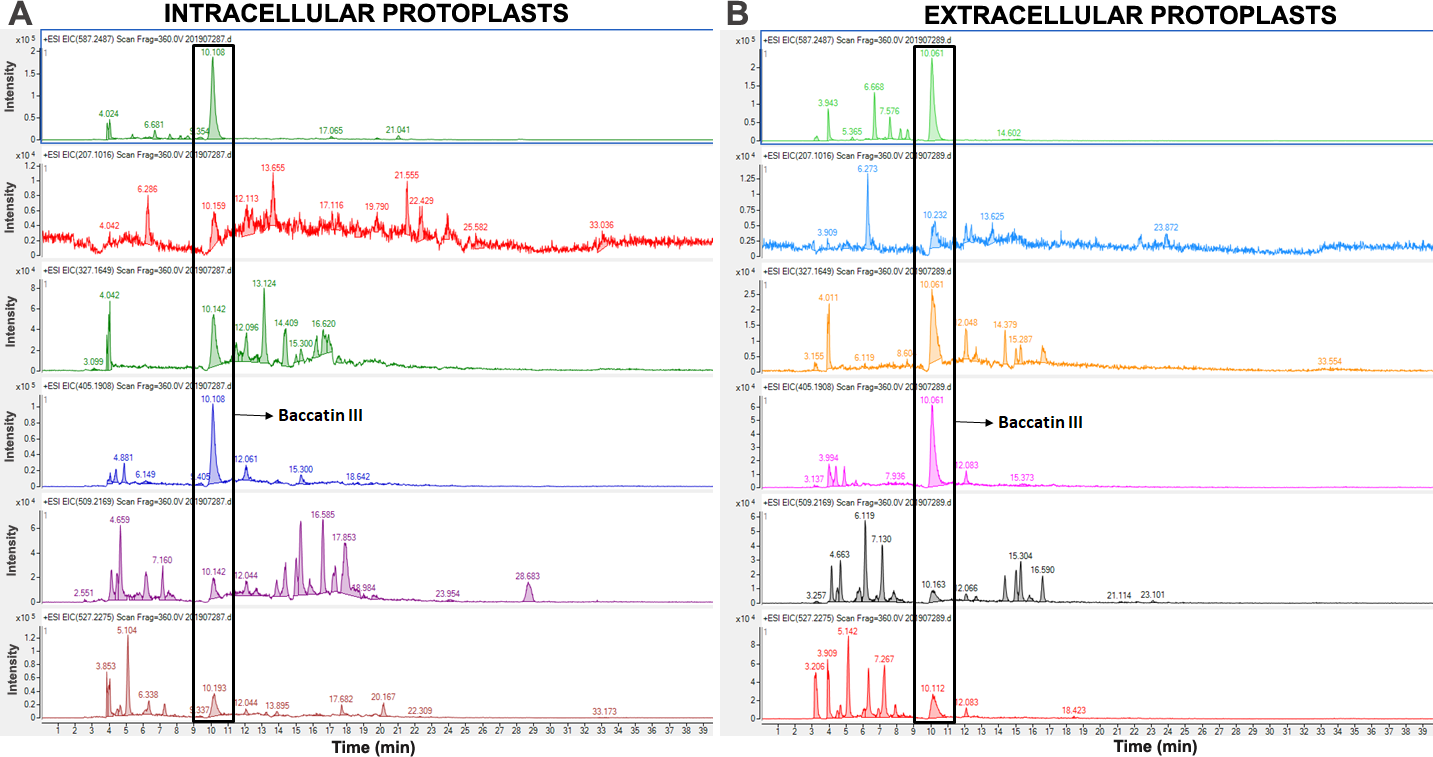


**Figure S8. HPLC-MS/MS chromatograms of intra (A) and extracellular (B) P. sativum protoplasts transiently transfected with the genes BAPT, DBTNBT and the candidate TB506 treated with 1 µM coronatine in the presence of baccatin III and ß-phenylalanine-CoA.** From above to below the chromatograms represent the different molecular ions corresponding with baccatin III (m/z: 587.248, 207.101, 327.164, 405.190, 509.216, and 527.227 which correspond to the retention times of 10.061, 10.061, 10.061, 10.061, 10.163 and 10.112 min, respectively).


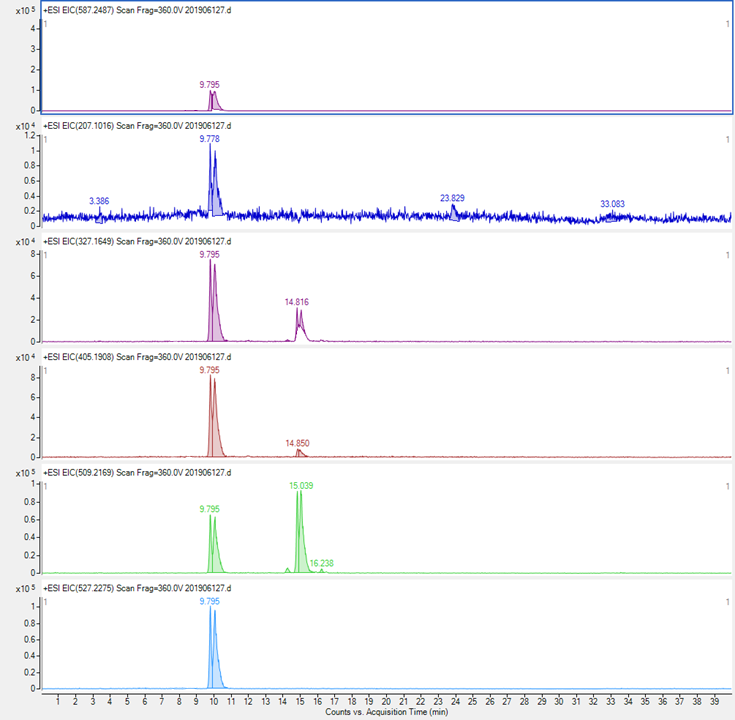


**Figure S9. HPLC-MS/MS chromatograms the standard solution of baccatin III.** From above to below the chromatograms represent the different molecular ions corresponding with baccatin III (m/z: 587.248, 207.101, 327.164, 405.190, 509.216 and 527.227 which correspond to the retention time of 10.10 min).

**
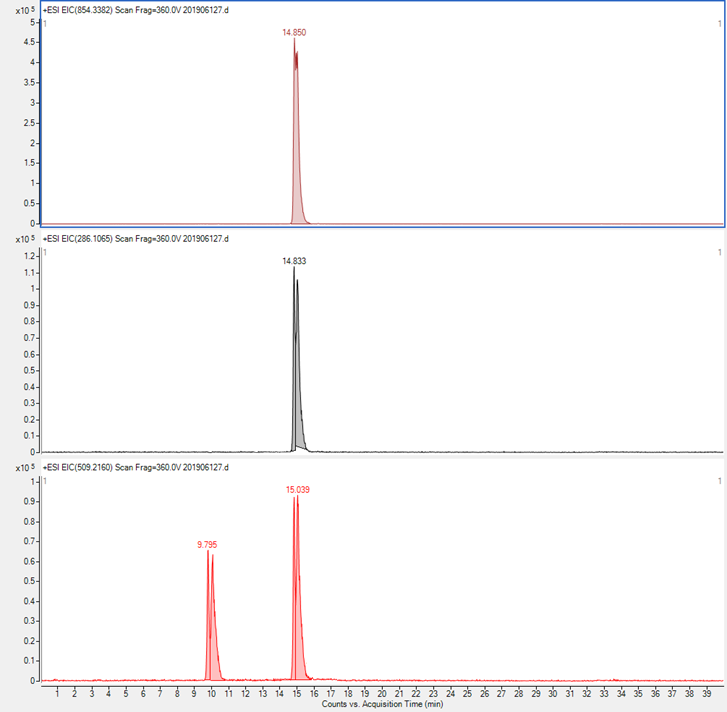
**

**Figure S10. HPLC-MS/MS chromatograms the standard solution of paclitaxel.** From above to below the chromatograms represent the different molecular ions corresponding with paclitaxel (m/z: 854.338, 286.106 and 509.2160 which correspond to the retention time of 14.992 min).


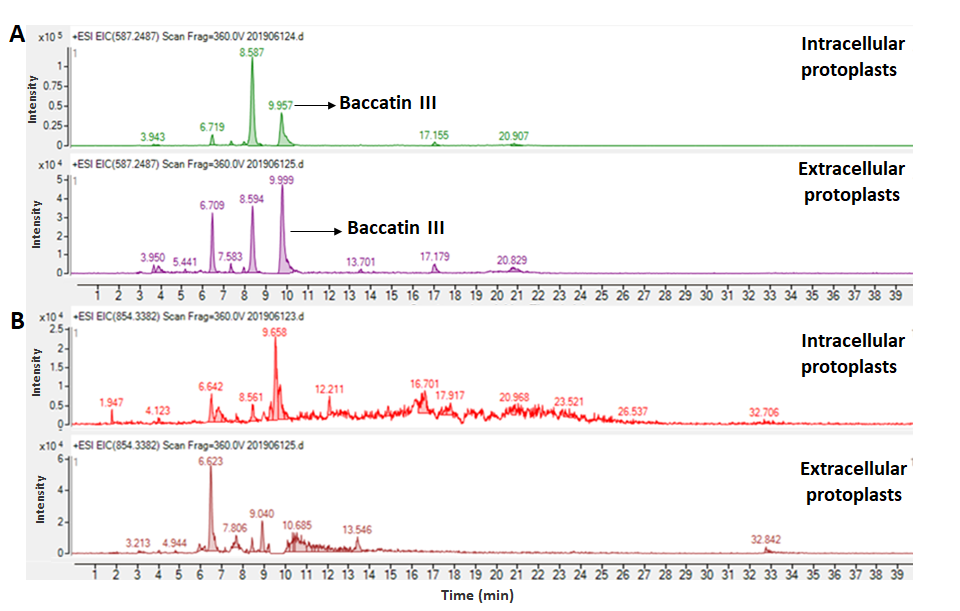


**Figure S11. HPLC-MS/MS analysis of control protoplasts transfected with the genes BAPT and DBTNBT treated with 1 µM coronatine in the presence of baccatin III and ß-phenylalanine-CoA.** (A) Identification of baccatin III in both inside and outside protoplasts of P. sativum at 72 h of incubation. (B) Paclitaxel was detected neither on the intracellular nor the extracellular sample of P. sativum protoplasts at 72 h of incubation.
