# Supplementary material for: A Novel Hydroxylation Step in the Taxane Biosynthetic Pathway: A New Approach to Paclitaxel Production by Synthetic Biology
Source: Front Bioeng Biotechnol. 2020 May 13;8:410. doi: 10.3389/fbioe.2020.00410 (PMC7247824; doi:10.3389/fbioe.2020.00410)
Supplement: Supplementary file 2 [file Table_1.pdf]

#	Template	Alignment Coverage	3D Model	Confidence	% i.d.	Template Information
1	c2hi4A_	Alignment	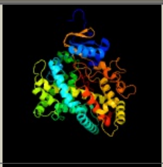	100.0	26	**PDB header:**oxidoreductase<br>**Chain:** A: **PDB Molecule:**cytochrome p450 1a2;<br>**PDBTitle:** crystal structure of human microsomal p450 1a2 in complex with alpha-2 naphthoflavone
2	c5ylwA_	Alignment	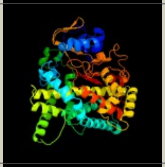	100.0	25	**PDB header:**oxidoreductase<br>**Chain:** A: **PDB Molecule:**ferruginol synthase;<br>**PDBTitle:** cyp76ah1 from salvia miltiorrhiza
3	d1nr6a_	Alignment	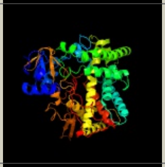	100.0	23	**Fold:**Cytochrome P450<br>**Superfamily:**Cytochrome P450<br>**Family:**Cytochrome P450
4	d2nnja1	Alignment	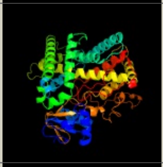	100.0	24	**Fold:**Cytochrome P450<br>**Superfamily:**Cytochrome P450<br>**Family:**Cytochrome P450
5	c3e4eA_	Alignment	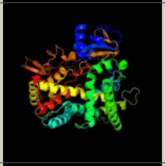	100.0	23	**PDB header:**oxidoreductase<br>**Chain:** A: **PDB Molecule:**cytochrome p450 2e1;<br>**PDBTitle:** human cytochrome p450 2e1 in complex with the inhibitor 4-2 methylpyrazole
6	d1po5a_	Alignment	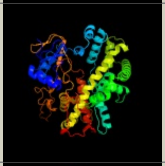	100.0	24	**Fold:**Cytochrome P450<br>**Superfamily:**Cytochrome P450<br>**Family:**Cytochrome P450
7	c5t6qA_	Alignment	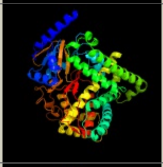	100.0	19	**PDB header:**oxidoreductase<br>**Chain:** A: **PDB Molecule:**cytochrome p450 4b1;<br>**PDBTitle:** structure of cytochrome p450 4b1 (cyp4b1) complexed with octane: an n-2 alkane and fatty acid omega-hydroxylase with a covalently bound heme
8	c3ebsA_	Alignment	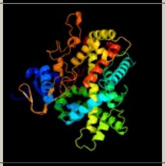	100.0	25	**PDB header:**oxidoreductase<br>**Chain:** A: **PDB Molecule:**cytochrome p450 2a6;<br>**PDBTitle:** human cytochrome p450 2a6 i208s/i300f/g301a/s369g in complex2 with phenacetin
9	c3k9vB_	Alignment	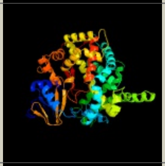	100.0	21	**PDB header:**oxidoreductase<br>**Chain:** B: **PDB Molecule:**1,25-dihydroxyvitamin d(3) 24-hydroxylase, mitochondrial;<br>**PDBTitle:** crystal structure of rat mitochondrial p450 24a1 s57d in complex with2 chaps
10	c6b82B_	Alignment	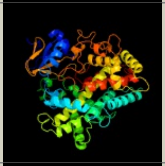	100.0	25	**PDB header:**oxidoreductase<br>**Chain:** B: **PDB Molecule:**cytochrome p450, family 17, subfamily a, polypeptide 1;<br>**PDBTitle:** zebra fish cyp-450 17a1 mutant abiraterone complex
11	d3czha1	Alignment	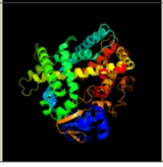	100.0	22	**Fold:**Cytochrome P450<br>**Superfamily:**Cytochrome P450<br>**Family:**Cytochrome P450

12	c4lxjA_	Alignment	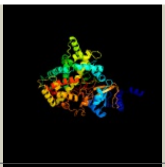	100.0	14	**PDB header:**oxidoreductase<br>**Chain:** A: **PDB Molecule:**lanosterol 14-alpha demethylase;<br>**PDBTitle:** saccharomyces cerevisiae lanosterol 14-alpha demethylase with2 lanosterol bound
13	d1r9oa_	Alignment	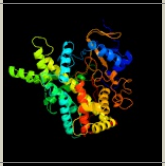	100.0	23	**Fold:**Cytochrome P450<br>**Superfamily:**Cytochrome P450<br>**Family:**Cytochrome P450
14	c4fdhA_	Alignment	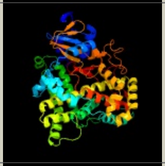	100.0	18	**PDB header:**oxidoreductase/oxidoreductase inhibitor<br>**Chain:** A: **PDB Molecule:**cytochrome p450 11b2, mitochondrial;<br>**PDBTitle:** structure of human aldosterone synthase, cyp11b2, in complex with2 fadrozole
15	c3na0B_	Alignment	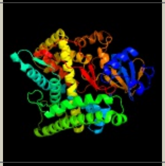	100.0	19	**PDB header:**oxidoreductase, electron transport<br>**Chain:** B: **PDB Molecule:**cholesterol side-chain cleavage enzyme, mitochondrial;<br>**PDBTitle:** crystal structure of human cyp11a1 in complex with 20,22-2 dihydroxycholesterol
16	d1tqna_	Alignment	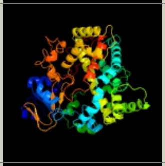	100.0	20	**Fold:**Cytochrome P450<br>**Superfamily:**Cytochrome P450<br>**Family:**Cytochrome P450
17	c2x2nB_	Alignment	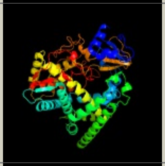	100.0	13	**PDB header:**oxidoreductase<br>**Chain:** B: **PDB Molecule:**lanosterol 14-alpha-demethylase;<br>**PDBTitle:** x-ray structure of cyp51 from trypanosoma brucei in complex2 with posaconazole in two different conformations
18	c2iagA_	Alignment	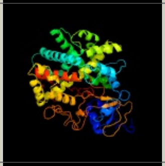	100.0	16	**PDB header:**isomerase<br>**Chain:** A: **PDB Molecule:**prostacyclin synthase;<br>**PDBTitle:** crystal structure of human prostacyclin synthase
19	d2ij2a1	Alignment	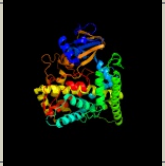	100.0	19	**Fold:**Cytochrome P450<br>**Superfamily:**Cytochrome P450<br>**Family:**Cytochrome P450
20	c3rukD_	Alignment	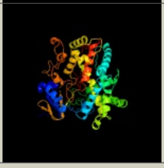	100.0	26	**PDB header:**oxidoreductase/oxidoreductase inhibitor<br>**Chain:** D: **PDB Molecule:**steroid 17-alpha-hydroxylase/17,20 lyase;<br>**PDBTitle:** human cytochrome p450 cyp17a1 in complex with abiraterone
21	c2f9qA_	Alignment	not modelled	100.0	23	**PDB header:**oxidoreductase<br>**Chain:** A: **PDB Molecule:**cytochrome p450 2d6;<br>**PDBTitle:** crystal structure of human cytochrome p450 2d6
22	c3g1qC_	Alignment	not modelled	100.0	15	**PDB header:**oxidoreductase<br>**Chain:** C: **PDB Molecule:**sterol 14-alpha-demethylase;<br>**PDBTitle:** crystal structure of sterol 14-alpha demethylase (cyp51) from2 trypanosoma brucei in ligand free state
23	c5fsaA_	Alignment	not modelled	100.0	17	**PDB header:**oxidoreductase<br>**Chain:** A: **PDB Molecule:**cyp51 variant1;<br>**PDBTitle:** crystal structure of sterol 14-alpha demethylase (cyp51) from a2 pathogenic yeast candida albicans in complex with the antifungal drug3 posaconazole
24	c6mcwA_	Alignment	not modelled	100.0	21	**PDB header:**oxidoreductase<br>**Chain:** A: **PDB Molecule:**cytochrome p450 51;<br>**PDBTitle:** crystal structure of the p450 domain of the cyp51-ferredoxin fusion2 protein from methylococcus capsulatus, complex with the detergent3 anapoe-x-114
25	c6a18A_	Alignment	not modelled	100.0	16	**PDB header:**oxidoreductase<br>**Chain:** A: **PDB Molecule:**cytochrome p450 90b1;<br>**PDBTitle:** crystal structure of cyp90b1 in complex with 1,6-hexandiol
26	c3juvA_	Alignment	not modelled	100.0	15	**PDB header:**oxidoreductase<br>**Chain:** A: **PDB Molecule:**lanosterol 14-alpha demethylase;<br>**PDBTitle:** crystal structure of human lanosterol 14alpha-demethylase (cyp51)
27	c3daxB_	Alignment	not modelled	100.0	16	**PDB header:**oxidoreductase<br>**Chain:** B: **PDB Molecule:**cytochrome p450 7a1;<br>**PDBTitle:** crystal structure of human cyp7a1
28	c2ve3A_	Alignment	not modelled	100.0	19	**PDB header:**oxidoreductase<br>**Chain:** A: **PDB Molecule:**putative cytochrome p450 120;<br>**PDBTitle:** retinoic acid bound cyanobacterial cyp120a1

29	c2rchA_	Alignment	not modelled	100.0	14	**PDB header:**lyase **Chain:** A: **PDB Molecule:**cytochrome p450 74a; **PDBTitle:** crystal structure of arabidopsis thaliana allene oxide synthase (aos,2 cytochrome p450 74a, cyp74a) complexed with 13(s)-hod at 1.85 a3 resolution
30	c3qz1D_	Alignment	not modelled	100.0	23	**PDB header:**oxidoreductase **Chain:** D: **PDB Molecule:**steroid 21-hydroxylase; **PDBTitle:** crystal structure of bovine steroid of 21-hydroxylase (p450c21)
31	c3danA_	Alignment	not modelled	100.0	14	**PDB header:**lyase **Chain:** A: **PDB Molecule:**cytochrome p450 74a2; **PDBTitle:** crystal structure of allene oxide synthase
32	c3pm0A_	Alignment	not modelled	100.0	22	**PDB header:**oxidoreductase **Chain:** A: **PDB Molecule:**cytochrome p450 1b1; **PDBTitle:** structural characterization of the complex between alpha-2 naphthoflavone and human cytochrome p450 1b1 (cyp1b1)
33	c5tl8A_	Alignment	not modelled	100.0	17	**PDB header:**oxidoreductase **Chain:** A: **PDB Molecule:**protein cyp51; **PDBTitle:** naegleria fowleri cyp51-posaconazole complex
34	c3eqmA_	Alignment	not modelled	100.0	14	**PDB header:**oxidoreductase **Chain:** A: **PDB Molecule:**cytochrome p450 19a1; **PDBTitle:** crystal structure of human placental aromatase cytochrome p450 in2 complex with androstenedione
35	c4r21B_	Alignment	not modelled	100.0	26	**PDB header:**oxidoreductase **Chain:** B: **PDB Molecule:**cytochrome p450 family 17 polypeptide 2; **PDBTitle:** zebra fish cytochrome p450 17a2 with progesterone
36	c3hf2A_	Alignment	not modelled	100.0	19	**PDB header:**oxidoreductase **Chain:** A: **PDB Molecule:**bifunctional p-450/nadph-p450 reductase; **PDBTitle:** crystal structure of the i401p mutant of cytochrome p450 bm3
37	c6fyjA_	Alignment	not modelled	100.0	15	**PDB header:**oxidoreductase **Chain:** A: **PDB Molecule:**fatty-acid peroxygenase; **PDBTitle:** cytochrome p450 peroxygenase cyp152k6 in complex with myristic acid
38	d2ciba1	Alignment	not modelled	100.0	17	**Fold:**Cytochrome P450 **Superfamily:**Cytochrome P450 **Family:**Cytochrome P450
39	c6n6qC_	Alignment	not modelled	100.0	22	**PDB header:**oxidoreductase **Chain:** C: **PDB Molecule:**cytochrome p450 (cyp102l1); **PDBTitle:** crystal structure of a cytochrome p450 (cyp102l1)
40	c6j94A_	Alignment	not modelled	100.0	18	**PDB header:**oxidoreductase **Chain:** A: **PDB Molecule:**protein lutein deficient 5, chloroplastic; **PDBTitle:** crystal structure of cyp97a3
41	c2q9fA_	Alignment	not modelled	100.0	21	**PDB header:**oxidoreductase **Chain:** A: **PDB Molecule:**cytochrome p450 46a1; **PDBTitle:** crystal structure of human cytochrome p450 46a1 in complex with2 cholesterol-3-sulphate
42	c3b99B_	Alignment	not modelled	100.0	18	**PDB header:**isomerase **Chain:** B: **PDB Molecule:**prostaglandin i2 synthase; **PDBTitle:** crystal structure of zebrafish prostacyclin synthase (cytochrome p4502 8a1) in complex with substrate analog u51605
43	c3el3A_	Alignment	not modelled	100.0	19	**PDB header:**oxidoreductase **Chain:** A: **PDB Molecule:**putative cytochrome p450; **PDBTitle:** distinct monooxygenase and farnesene synthase active sites2 in cytochrome p450 170a1
44	c4l54A_	Alignment	not modelled	100.0	18	**PDB header:**oxidoreductase **Chain:** A: **PDB Molecule:**terminal olefin-forming fatty acid decarboxylase; **PDBTitle:** structure of cytochrome p450 olet, ligand-free
45	c5yhjB_	Alignment	not modelled	100.0	15	**PDB header:**oxidoreductase **Chain:** B: **PDB Molecule:**cytochrome p450; **PDBTitle:** cytochrome p450ex alpha (cyp152n1) wild-type with myristic acid
46	d1izoa_	Alignment	not modelled	100.0	18	**Fold:**Cytochrome P450 **Superfamily:**Cytochrome P450 **Family:**Cytochrome P450
47	c6j85B_	Alignment	not modelled	100.0	16	**PDB header:**oxidoreductase **Chain:** B: **PDB Molecule:**nocardicin n-oxygenase; **PDBTitle:** crystal structure of hind apo
48	c4uylB_	Alignment	not modelled	100.0	14	**PDB header:**oxidoreductase **Chain:** B: **PDB Molecule:**14-alpha sterol demethylase; **PDBTitle:** crystal structure of sterol 14-alpha demethylase (cyp51b) from a2 pathogenic filamentous fungus aspergillus fumigatus in complex3 with vni
49	c5fyfA_	Alignment	not modelled	100.0	15	**PDB header:**oxidoreductase **Chain:** A: **PDB Molecule:**cytochrome p450; **PDBTitle:** structure of cyp153a from marinobacter aquaeolei
50	c3awmA_	Alignment	not modelled	100.0	17	**PDB header:**oxidoreductase **Chain:** A: **PDB Molecule:**fatty acid alpha-hydroxylase; **PDBTitle:** cytochrome p450sp alpha (cyp152b1) wild-type with palmitic acid
51	c2zbxA_	Alignment	not modelled	100.0	16	**PDB header:**oxidoreductase **Chain:** A: **PDB Molecule:**cytochrome p450-su1; **PDBTitle:** crystal structure of vitamin d hydroxylase cytochrome p4502 105a1 (wild type) with imidazole bound
52	c2z36A_	Alignment	not modelled	100.0	17	**PDB header:**oxidoreductase **Chain:** A: **PDB Molecule:**cytochrome p450 type compactin 3'',4''- **PDBTitle:** crystal structure of cytochrome p450 moxa from nonomuraea2 recticatena (cyp105)
53	c3lxiB_	Alignment	not modelled	100.0	13	**PDB header:**oxidoreductase **Chain:** B: **PDB Molecule:**cytochrome p450; **PDBTitle:** crystal structure of camphor-bound cyp101d1
54	c4xe3B_	Alignment	not modelled	100.0	17	**PDB header:**oxidoreductase **Chain:** B: **PDB Molecule:**cytochrome p-450; **PDBTitle:** olep, the cytochrome p450 epoxidase from streptomyces antibioticus2 involved in oleandomycin biosynthesis: functional

						analysis and3 crystallographic structure in complex with clotrimazole.
55	c4r1zB_	Alignment	not modelled	100.0	25	**PDB header:**oxidoreductase<br>**Chain:** B: **PDB Molecule:**cyp17a1 protein;<br>**PDBTitle:** zebra fish cytochrome p450 17a1 with abiraterone
56	c3tktA_	Alignment	not modelled	100.0	17	**PDB header:**oxidoreductase<br>**Chain:** A: **PDB Molecule:**cytochrome p450;<br>**PDBTitle:** crystal structure of cyp108d1 from novosphingobium aromaticivorans2 dsm12444
57	c4jbtB_	Alignment	not modelled	100.0	20	**PDB header:**oxidoreductase/substrate<br>**Chain:** B: **PDB Molecule:**cytochrome p450 monooxygenase;<br>**PDBTitle:** the 2.2 a crystal structure of cyp154c5 from nocardia farcinica in2 complex with androstenedione
58	c2y46B_	Alignment	not modelled	100.0	16	**PDB header:**oxidoreductase<br>**Chain:** B: **PDB Molecule:**p-450-like protein;<br>**PDBTitle:** structure of the mixed-function p450 mycg in complex with mycinamicin2 iv in c 2 2 21 space group
59	d1re9a_	Alignment	not modelled	100.0	12	**Fold:**Cytochrome P450<br>**Superfamily:**Cytochrome P450<br>**Family:**Cytochrome P450
60	d1z8oa1	Alignment	not modelled	100.0	17	**Fold:**Cytochrome P450<br>**Superfamily:**Cytochrome P450<br>**Family:**Cytochrome P450
61	c3a4hA_	Alignment	not modelled	100.0	15	**PDB header:**oxidoreductase<br>**Chain:** A: **PDB Molecule:**vitamin d hydroxylase;<br>**PDBTitle:** structure of cytochrome p450 vdh from pseudonocardia autotrophica2 (orthorhombic crystal form)
62	c2c6hB_	Alignment	not modelled	100.0	16	**PDB header:**oxidoreductase<br>**Chain:** B: **PDB Molecule:**cytochrome p450 monooxygenase;<br>**PDBTitle:** crystal structure of yc-17-bound cytochrome p450 pikc2 (cyp107l1)
63	c3tywC_	Alignment	not modelled	100.0	16	**PDB header:**oxidoreductase<br>**Chain:** C: **PDB Molecule:**putative cytochrome p450;<br>**PDBTitle:** crystal structure of cyp105n1 from streptomyces coelicolor a3(2)
64	c5y1iA_	Alignment	not modelled	100.0	17	**PDB header:**oxidoreductase<br>**Chain:** A: **PDB Molecule:**cytochrome p450;<br>**PDBTitle:** the crystal structure of gfsf
65	c4dxyA_	Alignment	not modelled	100.0	13	**PDB header:**oxidoreductase<br>**Chain:** A: **PDB Molecule:**cytochrome p450;<br>**PDBTitle:** crystal structures of cyp101d2 y96a mutant
66	d1gwia_	Alignment	not modelled	100.0	19	**Fold:**Cytochrome P450<br>**Superfamily:**Cytochrome P450<br>**Family:**Cytochrome P450
67	c6gk5A_	Alignment	not modelled	100.0	17	**PDB header:**oxidoreductase<br>**Chain:** A: **PDB Molecule:**cytochrome p450 cyp267b1 protein;<br>**PDBTitle:** crystal structure of cytochrome p450 cyp267b1 from sorangium2 cellulosum so ce56
68	c3ejdD_	Alignment	not modelled	100.0	16	**PDB header:**oxidoreductase/lipid transport<br>**Chain:** D: **PDB Molecule:**biotin biosynthesis cytochrome p450-like enzyme;<br>**PDBTitle:** crystal structure of p450bioi in complex with hexadec-9z-enoic acid2 ligated acyl carrier protein
69	c4l0eA_	Alignment	not modelled	100.0	16	**PDB header:**oxidoreductase<br>**Chain:** A: **PDB Molecule:**p450 monooxygenase;<br>**PDBTitle:** structure of p450sky (cyp163b3), a cytochrome p450 from skyllamycin2 biosynthesis (heme-coordinated expression tag)
70	c3wvsA_	Alignment	not modelled	100.0	16	**PDB header:**oxidoreductase<br>**Chain:** A: **PDB Molecule:**putative monooxygenase;<br>**PDBTitle:** crystal structure of cytochrome p450revi
71	c4z5pB_	Alignment	not modelled	100.0	16	**PDB header:**oxidoreductase<br>**Chain:** B: **PDB Molecule:**cytochrome p450 hydroxylase;<br>**PDBTitle:** crystal structure of the lnma cytochrome p450 hydroxylase from the2 leinamycin biosynthetic pathway of streptomyces atroolivaceus s-1403 at 1.9 a resolution
72	c2dkkA_	Alignment	not modelled	100.0	13	**PDB header:**oxidoreductase<br>**Chain:** A: **PDB Molecule:**cytochrome p450;<br>**PDBTitle:** structure/function studies of cytochrome p450 158a1 from streptomyces2 coelicolor a3(2)
73	c2fr7A_	Alignment	not modelled	100.0	18	**PDB header:**oxidoreductase<br>**Chain:** A: **PDB Molecule:**putative cytochrome p450;<br>**PDBTitle:** crystal structure of cytochrome p450 cyp199a2
74	c3e5kA_	Alignment	not modelled	100.0	16	**PDB header:**oxidoreductase<br>**Chain:** A: **PDB Molecule:**cytochrome p450 (cytochrome p450 hydroxylase);<br>**PDBTitle:** crystal structure of cyp105p1 wild-type 4-phenylimidazole complex
75	c5l1sA_	Alignment	not modelled	100.0	17	**PDB header:**oxidoreductase<br>**Chain:** A: **PDB Molecule:**pentalenolactone synthase;<br>**PDBTitle:** x-ray structure of f232l mutant of cytochrome p450 pntm with2 pentalenolactone f
76	c5nwsA_	Alignment	not modelled	100.0	16	**PDB header:**biosynthetic protein<br>**Chain:** A: **PDB Molecule:**saacmm;<br>**PDBTitle:** crystal structure of saacmm involved in actinomycin biosynthesis
77	c6hqdB_	Alignment	not modelled	100.0	15	**PDB header:**oxidoreductase<br>**Chain:** B: **PDB Molecule:**cytochrome p450;<br>**PDBTitle:** cytochrome p450-153 from pseudomonas sp. 19-rlim
78	c6j84A_	Alignment	not modelled	100.0	15	**PDB header:**oxidoreductase<br>**Chain:** A: **PDB Molecule:**cytochrome p-450;<br>**PDBTitle:** crystal structure of tleb with hydroxyl analog
79	d1odoa_	Alignment	not modelled	100.0	20	**Fold:**Cytochrome P450<br>**Superfamily:**Cytochrome P450<br>**Family:**Cytochrome P450
						**PDB header:**oxidoreductase

80	c3nv6A_	Alignment	not modelled	100.0	11	**Chain:** A: **PDB Molecule:**cytochrome p450; **PDBTitle:** crystal structure of camphor-bound cyp101d2
81	c2wivA_	Alignment	not modelled	100.0	18	**PDB header:**electron transport **Chain:** A: **PDB Molecule:**cytochrome p450-like protein xpla; **PDBTitle:** cytochrome-p450 xpla heme domain p21
82	c5foiB_	Alignment	not modelled	100.0	17	**PDB header:**oxidoreductase **Chain:** B: **PDB Molecule:**mycinamicin viii c21 methyl hydroxylase; **PDBTitle:** crystal structure of mycinamicin viii c21 methyl hydroxylase mycci2 from micromonospora griseorubida bound to mycinamicin viii
83	c4mm0B_	Alignment	not modelled	100.0	17	**PDB header:**oxidoreductase **Chain:** B: **PDB Molecule:**p450-like monooxygenase; **PDBTitle:** crystal structure analysis of the putative thioether synthase sgvp2 involved in the tailoring step of griseoviridin
84	c5ncbA_	Alignment	not modelled	100.0	12	**PDB header:**oxidoreductase **Chain:** A: **PDB Molecule:**cytochrome p450; **PDBTitle:** crystal structure of amycolatopsis cytochrome p450 gcoa in complex2 with guaiacol.
85	d1s1fa_	Alignment	not modelled	100.0	18	**Fold:**Cytochrome P450 **Superfamily:**Cytochrome P450 **Family:**Cytochrome P450
86	c4ubsA_	Alignment	not modelled	100.0	17	**PDB header:**oxidoreductase **Chain:** A: **PDB Molecule:**pentalenic acid synthase; **PDBTitle:** the crystal structure of cytochrome p450 105d7 from streptomyces2 avermitilis in complex with diclofenac
87	c3rwlA_	Alignment	not modelled	100.0	17	**PDB header:**oxidoreductase **Chain:** A: **PDB Molecule:**cytochrome p450 alkane hydroxylase 1 cyp153a7; **PDBTitle:** structure of p450pyr hydroxylase
88	d1jfba_	Alignment	not modelled	100.0	17	**Fold:**Cytochrome P450 **Superfamily:**Cytochrome P450 **Family:**Cytochrome P450
89	d1n40a_	Alignment	not modelled	100.0	17	**Fold:**Cytochrome P450 **Superfamily:**Cytochrome P450 **Family:**Cytochrome P450
90	d1cpta_	Alignment	not modelled	100.0	16	**Fold:**Cytochrome P450 **Superfamily:**Cytochrome P450 **Family:**Cytochrome P450
91	c5h1zA_	Alignment	not modelled	100.0	17	**PDB header:**hydrolase **Chain:** A: **PDB Molecule:**putative cyp alkane hydroxylase cyp153d17; **PDBTitle:** cyp153d17 from sphingomonas sp. pamc 26605
92	d1q5da_	Alignment	not modelled	100.0	17	**Fold:**Cytochrome P450 **Superfamily:**Cytochrome P450 **Family:**Cytochrome P450
93	c3r9cA_	Alignment	not modelled	100.0	19	**PDB header:**oxidoreductase **Chain:** A: **PDB Molecule:**cytochrome p450 164a2; **PDBTitle:** crystal structure of mycobacterium smegmatis cyp164a2 with econazole2 bound
94	c3bujA_	Alignment	not modelled	100.0	18	**PDB header:**metal binding protein **Chain:** A: **PDB Molecule:**calo2; **PDBTitle:** crystal structure of calo2
95	d1lfka_	Alignment	not modelled	100.0	16	**Fold:**Cytochrome P450 **Superfamily:**Cytochrome P450 **Family:**Cytochrome P450
96	c3abbA_	Alignment	not modelled	100.0	17	**PDB header:**oxidoreductase **Chain:** A: **PDB Molecule:**cytochrome p450 hydroxylase; **PDBTitle:** crystal structure of cyp105d6
97	c5hdiA_	Alignment	not modelled	100.0	15	**PDB header:**oxidoreductase **Chain:** A: **PDB Molecule:**cytochrome p450 144; **PDBTitle:** structural characterization of cyp144a1, a mycobacterium tuberculosis2 cytochrome p450
98	c2jjoA_	Alignment	not modelled	100.0	18	**PDB header:**oxidoreductase **Chain:** A: **PDB Molecule:**cytochrome p450 113a1; **PDBTitle:** structure of cytochrome p450 eryk in complex with its2 natural substrate erd
99	c6hqwA_	Alignment	not modelled	100.0	16	**PDB header:**oxidoreductase **Chain:** A: **PDB Molecule:**cytochrome p450; **PDBTitle:** cytochrome p450-153 from novosphingobium aromaticivorans
100	c4yzrA_	Alignment	not modelled	100.0	15	**PDB header:**oxidoreductase **Chain:** A: **PDB Molecule:**polyketide biosynthesis cytochrome p450 pkss; **PDBTitle:** bacillus subtilis 168 bacillaene polyketide synthase (pks) cytochrome2 p450 pkss
101	c5li8A_	Alignment	not modelled	100.0	16	**PDB header:**oxidoreductase **Chain:** A: **PDB Molecule:**putative cytochrome p450 126; **PDBTitle:** crystal structure of mycobacterium tuberculosis cyp126a1 in complex2 with ketoconazole
102	c5gweB_	Alignment	not modelled	100.0	15	**PDB header:**electron transport **Chain:** B: **PDB Molecule:**cytochrome p450; **PDBTitle:** cytochrome p450 crej
103	c5l90B_	Alignment	not modelled	100.0	18	**PDB header:**oxidoreductase **Chain:** B: **PDB Molecule:**cytochrome p450; **PDBTitle:** the crystal structure of substrate-free cyp109e1 from bacillus2 megaterium at 2.55 angstrom resolution
104	c6b11B_	Alignment	not modelled	100.0	15	**PDB header:**oxidoreductase **Chain:** B: **PDB Molecule:**20-oxo-5-o-mycaminosyltylactone 23-monooxygenase; **PDBTitle:** tylhi in complex with native substrate 23-deoxy-5-o-mycaminosyl-2 tylonolide (23-dmtl)
105	c2wm5A_	Alignment	not modelled	100.0	13	**PDB header:**oxidoreductase **Chain:** A: **PDB Molecule:**putative cytochrome p450 124; **PDBTitle:** x-ray structure of the substrate-free mycobacterium tuberculosis2 cytochrome p450 cyp124
						**PDB header:**oxidoreductase

106	c3p3oA_	Alignment	not modelled	100.0	16	**Chain:** A: **PDB Molecule:**cytochrome p450;<br>**PDBTitle:** crystal structure of the cytochrome p450 monooxygenase aurh (ntermii)2 from streptomyces thioluteus
107	c2xkrA_	Alignment	not modelled	100.0	14	**PDB header:**oxidoreductase<br>**Chain:** A: **PDB Molecule:**putative cytochrome p450 142;<br>**PDBTitle:** crystal structure of mycobacterium tuberculosis cyp142: a novel2 cholesterol oxidase
108	c2z3tD_	Alignment	not modelled	100.0	18	**PDB header:**oxidoreductase<br>**Chain:** D: **PDB Molecule:**cytochrome p450;<br>**PDBTitle:** crystal structure of substrate free cytochrome p450 stap2 (cyp245a1)
109	c2xbkA_	Alignment	not modelled	100.0	18	**PDB header:**oxidoreductase<br>**Chain:** A: **PDB Molecule:**pimd protein;<br>**PDBTitle:** x-ray structure of the substrate-bound cytochrome p450 pimd - a2 polyene macrolide antibiotic pimaricin epoxidase
110	c5cjeA_	Alignment	not modelled	100.0	16	**PDB header:**oxidoreductase<br>**Chain:** A: **PDB Molecule:**cytochrome p450 hydroxylase;<br>**PDBTitle:** structure of cyp107l2
111	c1t2bA_	Alignment	not modelled	100.0	15	**PDB header:**unknown function<br>**Chain:** A: **PDB Molecule:**p450cin;<br>**PDBTitle:** crystal structure of cytochrome p450cin complexed with its2 substrate 1,8-cineole
112	c6giiA_	Alignment	not modelled	100.0	15	**PDB header:**oxidoreductase<br>**Chain:** A: **PDB Molecule:**cytochrome p450;<br>**PDBTitle:** the crystal structure of tepidiphilus thermophilus p450 heme domain
113	c6g71A_	Alignment	not modelled	100.0	16	**PDB header:**oxidoreductase<br>**Chain:** A: **PDB Molecule:**cytochrome p450;<br>**PDBTitle:** structure of cyp1232a24 from arthrobacter sp.
114	c6ro8A_	Alignment	not modelled	100.0	12	**PDB header:**oxidoreductase<br>**Chain:** A: **PDB Molecule:**cytochrome p450 rhf;<br>**PDBTitle:** the crystal structure of acinetobacter radioresistens cyp116b5 heme2 domain
115	c4z5qA_	Alignment	not modelled	100.0	16	**PDB header:**oxidoreductase<br>**Chain:** A: **PDB Molecule:**cytochrome p450 hydroxylase;<br>**PDBTitle:** crystal structure of the lnmz cytochrome p450 hydroxylase from the2 leinamycin biosynthetic pathway of streptomyces atroolivaceus s-1403 at 1.8 a resolution
116	d1n97a_	Alignment	not modelled	100.0	20	**Fold:**Cytochrome P450<br>**Superfamily:**Cytochrome P450<br>**Family:**Cytochrome P450
117	c3dbgA_	Alignment	not modelled	100.0	19	**PDB header:**oxidoreductase<br>**Chain:** A: **PDB Molecule:**putative cytochrome p450;<br>**PDBTitle:** crystal structure of cytochrome p450 170a1 (cyp170a1) from2 streptomyces coelicolor
118	c2uvnB_	Alignment	not modelled	100.0	14	**PDB header:**oxidoreductase<br>**Chain:** B: **PDB Molecule:**cytochrome p450 130;<br>**PDBTitle:** crystal structure of econazole-bound cyp130 from mycobacterium2 tuberculosis
119	c6f0bA_	Alignment	not modelled	100.0	14	**PDB header:**oxidoreductase<br>**Chain:** A: **PDB Molecule:**cytochrome p450 monooxygenase;<br>**PDBTitle:** cytochrome p450 txtc employs substrate conformational switching for2 sequential aliphatic and aromatic thaxtomin hydroxylation
120	c5hh3C_	Alignment	not modelled	100.0	18	**PDB header:**oxidoreductase<br>**Chain:** C: **PDB Molecule:**oxya protein;<br>**PDBTitle:** oxya from actinoplanes teichomyceticus
